# Wnt/β-catenin signaling regulates adipose tissue lipogenesis and adipocyte-specific loss is rigorously defended by neighboring stromal-vascular cells

**DOI:** 10.1016/j.molmet.2020.101078

**Published:** 2020-09-09

**Authors:** Devika P. Bagchi, Akira Nishii, Ziru Li, Jennifer B. DelProposto, Callie A. Corsa, Hiroyuki Mori, Julie Hardij, Brian S. Learman, Carey N. Lumeng, Ormond A. MacDougald

**Affiliations:** 1 Department of Molecular and Integrative Physiology, University of Michigan Medical School, Ann Arbor, MI, USA; 2 Department of Pediatrics and Communicable Diseases, University of Michigan Medical School, Ann Arbor, MI, USA; 3 Department of Microbiology and Immunology, University of Buffalo, Buffalo, NY, USA; 4 Division of Metabolism, Endocrinology, and Diabetes, Department of Internal Medicine, University of Michigan Medical School, Ann Arbor, MI, USA

**Keywords:** β-catenin, Wnt signaling, Adipocyte, Adipose tissue, Metabolism, Lipogenesis, *β-cat*^−/−^, adipocyte-specific β-catenin knockout, BAT, brown adipose tissue, DNL, *de novo* lipogenesis, eWAT, epididymal white adipose tissue, HFD, high-fat diet, iWAT, inguinal white adipose tissue, LRP, low-density lipoprotein receptor-related protein, MSC, mesenchymal stem cell, NCD, normal chow diet, pWAT, perirenal white adipose tissue, sEV, small extracellular vesicle, SVC, stromal-vascular cell, SVF, stromal-vascular fraction, TAG, triacylglycerol, TCF/LEF, T-cell factor/lymphoid enhancer-binding factor, WAT, white adipose tissue

## Abstract

**Objective:**

Canonical Wnt/β-catenin signaling is a well-studied endogenous regulator of mesenchymal cell fate determination, promoting osteoblastogenesis and inhibiting adipogenesis. However, emerging genetic evidence in humans links a number of Wnt pathway members to body fat distribution, obesity, and metabolic dysfunction, suggesting that this pathway also functions in adipocytes. Recent studies in mice have uncovered compelling evidence that the Wnt signaling pathway plays important roles in adipocyte metabolism, particularly under obesogenic conditions. However, complexities in Wnt signaling and differences in experimental models and approaches have thus far limited our understanding of its specific roles in this context.

**Methods:**

To investigate roles of the canonical Wnt pathway in the regulation of adipocyte metabolism, we generated adipocyte-specific β-catenin (*β-cat*) knockout mouse and cultured cell models. We used RNA sequencing, ChIP sequencing, and molecular approaches to assess expression of Wnt targets and lipogenic genes. We then used functional assays to evaluate effects of β-catenin deficiency on adipocyte metabolism, including lipid and carbohydrate handling. In mice maintained on normal chow and high-fat diets, we assessed the cellular and functional consequences of adipocyte-specific β-catenin deletion on adipose tissues and systemic metabolism.

**Results:**

We report that in adipocytes, the canonical Wnt/β-catenin pathway regulates *de novo* lipogenesis (DNL) and fatty acid monounsaturation. Further, β-catenin mediates effects of Wnt signaling on lipid metabolism in part by transcriptional regulation of *Mlxipl* and *Srebf1*. Intriguingly, adipocyte-specific loss of β-catenin is sensed and defended by CD45^-^/CD31^-^ stromal cells to maintain tissue-wide Wnt signaling homeostasis in chow-fed mice. With long-term high-fat diet, this compensatory mechanism is overridden, revealing that β-catenin deletion promotes resistance to diet-induced obesity and adipocyte hypertrophy and subsequent protection from metabolic dysfunction.

**Conclusions:**

Taken together, our studies demonstrate that Wnt signaling in adipocytes is required for lipogenic gene expression, *de novo* lipogenesis, and lipid desaturation. In addition, adipose tissues rigorously defend Wnt signaling homeostasis under standard nutritional conditions, such that stromal-vascular cells sense and compensate for adipocyte-specific loss. These findings underscore the critical importance of this pathway in adipocyte lipid metabolism and adipose tissue function.

## Highlights

• Wnt/β-catenin signaling regulates lipogenesis and lipid unsaturation in adipocytes.
• Effects on lipogenesis are mediated in part by the regulation of Mlxipl and Srebf1.
• Loss of adipocyte β-catenin is defended by CD45^-^/CD31^-^ cells in chow-fed mice.
• *β-cat*^−/−^ mice are protected from diet-induced obesity and metabolic dysfunction.

## Graphical abstract

**Figure:**
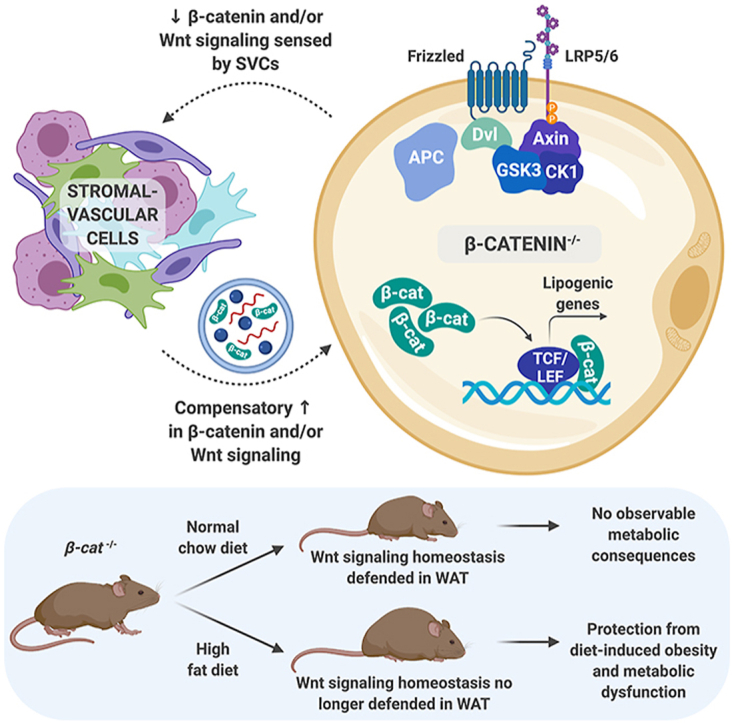

## 1 Introduction

Canonical Wnt/β-catenin signaling is an evolutionarily conserved pathway with well-established and diverse roles in cell proliferation and differentiation [1,2]. Binding of canonical Wnts to their membrane-spanning frizzled receptors and low-density lipoprotein receptor-related protein (LRP) co-receptors activates a complex intracellular signaling cascade that results in the stabilization and accumulation of free cytosolic β-catenin [3,4]. β-catenin can then translocate to the nucleus to bind to and coactivate DNA-binding T-cell factor/lymphoid enhancer-binding factor (TCF/LEF) proteins, thereby regulating Wnt target gene transcription [2,5,6].

Wnt signaling through the β-catenin-dependent pathway has been shown to have profound effects on mesenchymal cell fate determination and differentiation. Although this pathway is also involved in myogenesis and chondrogenesis [[7], [8], [9]], its differential regulatory roles in adipogenesis and osteogenesis have been particularly well-studied. Indeed, stabilization of canonical signaling by enforced expression of Wnt3a, Wnt6, Wnt10a, Wnt10b, or a β-catenin stable mutant in multipotent progenitor cells inhibits adipogenesis and promotes osteoblastogenesis [[10], [11], [12], [13], [14]]. Further, activation of canonical Wnt signaling in preadipocytes by enforced expression of Wnt1, Wnt10b, or a dominant-stable form of β-catenin, or pharmacological inhibition of glycogen synthase kinase 3, blocks adipogenesis by suppressing induction of the master adipogenic transcription factors PPARγ and C/EBPα [[15], [16], [17]]. In contrast, knockdown experiments in preadipocytes demonstrate that of the 19 Wnts, endogenous expression of Wnt6, Wnt10a, and Wnt10b contributes significantly to inhibition of adipogenesis [10]. Further, treatment of preadipocytes with soluble Wnt inhibitors sFRP1 or sFRP2 or overexpression of negative regulators of the canonical pathway, such as Axin or dominant-negative TCF4, results in spontaneous adipogenic differentiation [18,19]. Enforced expression of the β-catenin antagonist Chibby induces adipogenesis, whereas loss of Chibby upregulates β-catenin transcriptional activity and inhibits adipogenic differentiation [20,21]. Taken together, this significant body of work demonstrates that endogenous Wnt/β-catenin signaling is a critical repressor of adipocyte differentiation.

Although Wnt signaling is an important regulator of adipose tissue development [22,23], increasing genetic evidence also links this pathway to adiposity, body fat distribution, and metabolic dysfunction in humans. For example, missense variations in canonical *WNT10B* correlate with increased risk of type 2 diabetes [24], whereas polymorphisms in the Wnt inhibitor *SFRP5* locus are associated with decreased adiposity in men [25]. In addition, common variants in *RSPO3* and *ZNRF3* have been implicated in increased waist-to-hip ratios [[26], [27], [28]]. Patients harboring loss-of-function mutations in Wnt co-receptors *LRP5* and *LRP6* are more likely to suffer from impaired glucose homeostasis, coronary disease, and osteoporosis [29,30]; in contrast, gain-of-function mutations in *LRP5* are associated with increased adiposity and osteosclerosis [31]. Nonsense mutations in *LGR4*, a protein involved in stability of the Wnt co-receptor complex, correlate with reduced adiposity and impaired bone formation and remodeling [32], whereas patients with gain-of-function *LGR4* mutations are predisposed to increased visceral adiposity [33]. Perhaps most striking, genome-wide association studies across a broad range of ethnic populations have strongly linked polymorphisms in the canonical Wnt transcriptional activator *TCF7L2* to increased susceptibility to type 2 diabetes [[34], [35], [36]]. Additionally, during the course of our work, Chen et al. reported novel gain-of-function mutations in *CTNNB1* (β-catenin) that are associated with altered body fat distribution and predisposition to obesity [37]. These studies provide compelling genetic evidence for distinct roles of canonical Wnt/β-catenin signaling in body fat distribution and systemic metabolism.

Many recent studies in mice have investigated Wnt signaling in mature adipocytes, but its functional roles in this context remain unclear. For example, enforced expression of Wnt10b from the FABP4 promoter or stabilization of Wnt signaling through global deletion of the Wnt inhibitor secreted frizzled-related protein 5 (SFRP5) provides protection from diet-induced obesity [22,23,38]. In contrast, adipocyte-specific *Tcf7l2* deletion in obese mice results in increased adipocyte hypertrophy and impaired glucose homeostasis [39]. Our lab has demonstrated that loss of Wntless, and thus signaling by adipocyte-derived Wnts, protects mice from diet-induced obesity and metabolic dysfunction [40]. Consistent with these findings, Chen et al. recently reported that β-catenin knockout mice are resistant to diet-induced obesity and exhibit improved glucose homeostasis and insulin sensitivity compared to control counterparts [37]. Taken together, these reports demonstrate that Wnt signaling plays an important role in mature adipocytes, particularly under obesogenic conditions. However, additional studies are required to unravel the detailed mechanisms by which different Wnt pathway members contribute to metabolic function of terminally-differentiated cells. To this end, we investigated the roles of β-catenin, the central protein in the canonical Wnt pathway, specifically in adipocytes.

Herein, we report for the first time that β-catenin is a key regulator of adipocyte *de novo* lipogenesis (DNL) and fatty acid desaturation. Although chow-fed adipocyte-specific β-catenin knockout mice do not exhibit an overt phenotype, deeper investigation reveals a striking phenomenon in which surrounding stromal-vascular cells (SVC) dramatically upregulate β-catenin production and return mRNA and/or protein back to deficient adipocytes, such that knockout cells have sustained expression of β-catenin despite complete genomic deletion. Thus, Wnt signaling within and between cell types is monitored in white adipose tissue (WAT), and loss of β-catenin in adipocytes is compensated for by CD45^-^/CD31^-^ stromal cells to maintain whole-tissue Wnt pathway homeostasis under standard nutritional conditions. This compensatory mechanism is overridden by diet-induced obesity such that high-fat diet (HFD)-fed adipocyte-specific β-catenin knockout mice exhibit decreased adiposity and improved glucose homeostasis and hepatosteatosis. These findings underscore the critical importance of the canonical Wnt/β-catenin pathway in adipocyte lipid metabolism and adipose tissue function.

## 2 Materials and methods

### 2.1 Animals

*Ctnnb1*^fl/fl^ mice (hereby referred to as *β-cat*^fl/fl^) (#004152, Jackson Laboratory, Ellsworth, ME, USA) harboring loxP sites flanking exons 2 to 6 were crossed with adiponectin (*Adipoq*)-Cre mice (#028020, Jackson Laboratory, Ellsworth, ME, USA) to generate *β-cat*^fl/fl^ or *β-cat*^−/−^ mice. All animals were housed in a 12-h light/12-h dark cycle with free access to food and water. Mice used in HFD studies were fed rodent diet with 60 kcal% from fat (#12492, Research Diets, New Brunswick, NJ, USA). All animal studies were approved by and conducted in compliance with policies of the University of Michigan Institutional Animal Care and Use Committee. Daily care of mice was overseen by the Unit for Laboratory Animal Medicine at the University of Michigan.

### 2.2 Body composition

Lean, fat, and free fluid masses were measured by a Bruker Minispec LF90II NMR (Bruker, Billerica, MA, USA) at the University of Michigan Mouse Metabolic Phenotyping Center.

### 2.3 Glucose and insulin tolerance tests

For glucose tolerance tests, mice were fasted for 16 h and then given glucose (1 mg/kg body weight) via intraperitoneal injection. For insulin tolerance tests, mice were fasted for 6 h and then administered insulin (Eli Lilly, Indianapolis, IN, USA) via intraperitoneal injection. Chow-fed mice received 0.5 U insulin/kg body weight, whereas HFD-fed mice were given 1.0 U insulin/kg body weight. Glucose concentrations were monitored in blood collected from the tail vein at 0, 15, 30, 60, and 120 min after injection using a glucometer and Contour Next blood glucose strips (Bayer AG, Leverkusen, Germany).

### 2.4 *In vivo* lipolysis

Mice were administered an intraperitoneal injection of saline as a control or isoproterenol (10 mg/kg body weight) to stimulate lipolysis. Blood was collected from the tail vein immediately prior to and 15, 30, 60, and 120 min after injection. Blood was allowed to coagulate on ice for 2 h. After centrifugation at 2,000×*g* for 20 min at 4 °C, serum was transferred to a new tube. Serum glycerol concentrations were measured by colorimetric assays (Sigma–Aldrich, St. Louis, MO, USA).

### 2.5 Serum measurements

Blood was collected from the tail vein or by cardiac puncture at the time of sacrifice. After coagulation on ice for 2 h and centrifugation at 2,000×*g* for 20 min at 4 °C, separated serum was transferred to a new tube and stored at −80 °C until use. Serum insulin and adiponectin concentrations were measured by ELISA (Crystal Chem USA, Elk Grove, IL, USA, and R&D Systems Inc., Minneapolis, MN, USA, respectively). Colorimetric assays were used to estimate triacylglycerol (TAG) and total cholesterol levels (Cayman Chemical, Ann Arbor, MI, USA, and Abcam, Cambridge, UK, respectively).

### 2.6 Adipocyte and stromal-vascular cell fractionation

Epididymal WAT (eWAT) and inguinal WAT (iWAT) depots were excised from mice as previously described [41,42]. WAT depots were minced with scissors and digested in 2 mg/ml collagenase type I (Worthington Biochemical, Lakewood, NJ, USA) in Krebs-Ringer-HEPES (KRH; pH 7.4) buffer containing 3% fatty acid-free bovine serum albumin (BSA; Gold Biotechnology, St. Louis, MO, USA), 1 g/L glucose, and 500 nM adenosine for 1 h at 37 °C with shaking (600 rpm). Buoyant adipocytes were separated from the stromal-vascular fraction (SVF) by filtering cell suspensions through 100 μm cell strainers and then centrifuging at 100×*g* for 8 min. Fractions were washed twice with KRH buffer containing 3% fatty acid-free BSA, 1 g/L glucose, and 500 nM adenosine. For immunoblotting samples, fractions were then washed once with KRH buffer containing 0.5% fatty acid-free BSA, 1 g/L glucose, and 500 nM adenosine.

### 2.7 Fluorescence-activated cell sorting of stromal-vascular cell sub-populations

Excised eWAT was digested at 37 °C in RPMI medium (Thermo Fisher Scientific, Waltham, MA, USA) containing 0.5% BSA and 1 mg/ml type II collagenase for 30 min with shaking. To ensure sufficient cell numbers, three mice were combined per sample and three samples were analyzed per genotype. The SVF was separated from buoyant adipocytes by differential centrifugation following filtration through 100 μm cell strainers. Cells were stained with anti-CD45-PE (clone 30-F11; eBioscience, San Diego, CA, USA) and anti-CD31-APC (clone 390; eBioscience, San Diego, CA, USA) antibodies prior to flow cytometry analysis. Fluorescence-activated cell sorting was performed on a BD FACSAria III cell sorter (BD Biosciences, San Jose, CA, USA) and the data were processed and analyzed using FlowJo software (BD Biosciences, San Jose, CA, USA). Sorted cells were pelleted by centrifugation at 500×*g* for 5 min, and RNA was subsequently isolated for evaluation of gene expression.

### 2.8 Histology

After harvest, soft tissues were fixed overnight in 10% neutral-buffered formalin at 4 °C. Bones were fixed for 24 h in 10% neutral buffered formalin, rinsed with water, and then decalcified for 14 days in 14% EDTA at pH 7.4. Tissues were then processed, paraffin-embedded, and sectioned at 5 μm thickness. Sections were stained with hematoxylin and eosin as previously described [43] and subsequently imaged using a Zeiss inverted microscope at 100x or 200x magnification as indicated.

### 2.9 Cell culture

Primary mesenchymal stem cells (MSC) were isolated from outer ears of wild-type C57BL/6J (Jackson Laboratory, Bar Harbor, ME, USA) or *β-cat*^fl/fl^ mice as previously described [38,44] and cultured at 37 °C in 5% CO_2_. Sub-confluent MSCs were maintained in DMEM:F12 medium (Thermo Fisher Scientific, Waltham, MA, USA) containing 10% fetal bovine serum (FBS; Sigma–Aldrich, St. Louis, MO, USA) and supplemented with 10 ng/ml recombinant basic fibroblast growth factor (PeproTech Inc., Rocky Hill, NJ, USA). Adipogenesis was induced two days post–confluence with 5 μg/ml insulin, 5 μM rosiglitazone, 1 μM dexamethasone, and 0.5 mM methylisobutylxanthine in DMEM:F12 containing 10% FBS. Cells were fed fresh DMEM:F12 medium containing 10% FBS, 5 μg/ml insulin, and 5 μM rosiglitazone from days 2–4 of differentiation. For the remainder of differentiation, cells were maintained in DMEM:F12 containing 10% FBS. Oil Red O staining was used to visualize accumulation of neutral lipids as previously described [45]. Colorimetric assay (Cayman Chemical, Ann Arbor, MI, USA) was used to quantify total TAG accumulation per well. For lipid composition analyses, cells were differentiated in DMEM:F12 containing 10% charcoal-stripped FBS (Sigma–Aldrich, St. Louis, MO, USA) from days 6–12. As indicated, confluent MSCs or mature adipocytes were treated with 3 μM CHIR99021 (Cayman Chemical, Ann Arbor, MI, USA) or 20 ng/ml recombinant Wnt3a (R&D Systems Inc., Minneapolis, MN, USA) for 4 h prior to lysis and RNA isolation.

### 2.10 Genetic recombination in cultured cells

To induce gene deletion in preadipocytes, *β-cat*^fl/fl^ precursors were treated at ∼30% confluence with adenoviral green fluorescent protein (GFP) or adenoviral Cre recombinase (3 × 10^9^ viral particles/ml) in serum-free DMEM:F12 supplemented with 10 ng/ml recombinant basic fibroblast growth factor for 24 h. Preadipocytes were analyzed once they reached confluence. To induce gene recombination in adipocytes, *β-cat*^fl/fl^ cells were treated with adenoviral GFP or adenoviral Cre recombinase (1 × 10^10^ viral particles/ml) in serum-free DMEM:F12 from days 4–6 of differentiation. Adipocytes were then analyzed on day 12 of differentiation. Adenoviruses were obtained from the University of Michigan Vector Core. PCR with a three-primer system was used to confirm genetic recombination. Primer sequences were as follows: P1, AAGGTAGAGTGATGAAAGTTGTT; P2, CACCATGTCCTCTGTCTATTC; P3, TACACTATTGAATCACAGGGACTT; floxed band: 324 bp; and recombined band: 500 bp.

### 2.11 ChREBP or SREBP1c overexpression in cultured cells

*β-cat*^fl/fl^ and *β-cat*^−/−^ adipocytes were treated with adenoviral GFP, adenovirus overexpressing ChREBP, or adenovirus overexpressing SREBP1c (1 × 10^5^ viral particles/ml) in DMEM:F12 containing 10% FBS from days 9–12 of differentiation. Adipocytes were then analyzed on day 12 to evaluate DNL enzyme expression. Adenoviruses were obtained from Vector Biolabs (Vector Biolabs, Malvern, PA, USA).

### 2.12 De novo lipogenesis (DNL) assay

Prior to evaluation of DNL, cultured adipocytes were incubated in fresh serum-free DMEM:F12 medium overnight. To measure DNL, cells were then incubated in fresh DMEM:F12 medium (with 0.5 mM sodium pyruvate, 0.5 mM l-glutamine, 2.5 mM glucose, and 1% fatty acid-free BSA) containing 5 μM sodium acetate and 0.5 μCi [^14^C]-acetate (PerkinElmer, Waltham, MA, USA) for 2, 4, or 8 h at 37 °C. Following the indicated incubation times, cells were harvested and lipids extracted for analysis by scintillation counting and thin-layer chromatography.

### 2.13 Lipid extraction for analyses by gas chromatography

Lipids were extracted from cultured adipocytes as previously described [40]. Briefly, cells were washed twice with PBS and then collected in 500 μl of a 1:2.5 methanol/water mixture. Cell suspensions were then transferred to clean borosilicate glass tubes. Wells were rinsed with 500 μl of 1:2.5 methanol/water mixture and volumes were transferred to the glass tubes. After adding 750 μl chloroform and 375 μl 0.9% NaCl, tubes were vortexed vigorously and centrifuged at 2,500 rpm for 20 min at 4 °C. Lower organic chloroform layers containing total lipids were transferred to clean glass tubes and stored at −20 °C until use.

### 2.14 Fatty acid composition by gas chromatography

Fatty acids within extracted lipids were derivatized into their methyl esters by trans-esterification with boron trifluoride-methanol as previously described [46]. The derivatized methyl esters were re-dissolved in a small volume of hexane and purified by thin-layer chromatography using n-hexane-diethyl ether-acetic acid (50:50:2, v/v/v) as the developing solvent. Plates were dried and sprayed with Premuline after development. Products were identified under ultraviolet light by comparison to the retention flow of a methyl heptadecanoate (C17:0) standard (retention flow, 0.67) applied side by side on the same plate. Methyl esters were extracted from thin-layer chromatography powder with diethyl ether, concentrated under nitrogen, and re-dissolved in 100 μl hexane. Fatty acid compositions of lipids were analyzed by gas chromatography (GC) as follows: FAMEs analysis was conducted with a 1 μl sample injection on an Agilent GC machine model 6890N equipped with a flame ionization detector, auto sampler, and ChemStation software for data analysis. An Agilent HP 88 30 m GC column with a 0.25 mm inner diameter and 0.20 mm thick film was used, with hydrogen as a carrier gas and nitrogen as a makeup gas. Analyses were carried out with a temperature programming of 125–220 °C. Fatty acid components within unknown samples were identified with respect to the retention times of authentic standard methyl ester mixtures run side by side. Fatty acid components were quantified with respect to a known amount of internal standard added and the calibration ratio derived from each fatty acid of a standard methyl esters mixture and methyl heptadecanoate internal standard. The coefficient of variation for GC analyses was 2.3–3.7%.

### 2.15 Quantitative RT-PCR

Total RNA was isolated and purified from cultured cells or frozen tissue using RNA STAT-60 (Tel-Test, Alvin, TX, USA) according to the manufacturer's instructions. M-MLV Reverse Transcriptase (Invitrogen, Carlsbad, CA, USA) was used to reverse-transcribe 1 μg RNA to cDNA. qRT-PCR was performed using qPCRBIO SyGreen Mix (Innovative Solutions, Beverly Hills, MI, USA) on a StepOnePlus System (Applied Biosystems, Foster City, CA, USA). All primers were validated with cDNA titration curves prior to use; qPCR product specificities were confirmed by melting curve analysis and gel electrophoresis. Gene expression was calculated using a cDNA titration curve within each plate and subsequently normalized to peptidylprolyl isomerase A (PPIA) mRNA expression. The qPCR primer sequences are included in Supplemental Table 1.

### 2.16 RNA sequencing (RNA-seq) analyses

Confluent *β-cat*^fl/fl^ and *β-cat*^−/−^ preadipocytes and terminally-differentiated adipocytes were treated with 20 ng/ml recombinant Wnt3a (R&D Systems Inc., Minneapolis, MN, USA) or vehicle for 4 h prior to lysis and RNA isolation (n = 4 per group). After DNase treatment, samples were submitted to the University of Michigan Advanced Genomics Core for quality control, library preparation, and sequencing on an Illumina Hi-Seq platform. Read files for each sample were subsequently downloaded and collated into a single FASTQ file. FastQC (version 0.11.30) was used to evaluate the quality of raw read data and identify problematic features (inappropriate GC content, over-represented sequences, and/or low-quality scores). Alignment, differential expression analyses, and post-analysis diagnostics were conducted using the Tuxedo Suite software package. Briefly, TopHat (version 2.0.13) and Bowtie2 (version 2.2.1) were used to align reads to the UCSC reference genome. A second quality control round was performed post-alignment using FastQC to ensure that only high-quality data were used for gene expression quantitation and differential expression analyses. Differential expression analyses were performed using two independent methods, Cufflinks/CuffDiff and HTSeq/DESeq2, using UCSC build mm10 as the reference genome sequence. Pathway analyses were conducted on ranked lists of log2 fold change using Gene Set Enrichment Analysis (GSEA) software v4.0.3 and the Broad Institute Molecular Signature Database (MSigDB) [47]. Prior to running the analyses, mouse gene symbols were remapped to human ortholog symbols using chip annotation files. Mouse genes that did not have equivalent human orthologs were excluded from the analyses. Output from DESeq2 analyses was used to generate lists of genes ranked by the metric -log10 FDR ∗ log2 fold change. The resulting lists were run through pre-ranked GSEA using the Molecular Signatures Database v7.1 (H, hallmark gene sets). Enriched pathways were defined by an FDR <0.05. Normalized enrichment score (NES) is the primary metric from GSEA for evaluating the magnitude of differentially expressed pathways. Pathway impact analyses were conducted on AdvaitaBio's iPathwayGuide [48] to identify enriched KEGG pathways. The ggplot2 package in R was used to further visualize enriched pathways.

### 2.17 Chromatin immunoprecipitation (ChIP-seq) analyses

ChIP-seq data of Tcf7l2 binding sites in *Tcf7l2*^fl/fl^ and *Tcf7l2*^−/−^ cultured adipocytes were previously generated [39] and are publicly available. Data were obtained from Gene Expression Omnibus (GEO accession: GSE129403). The Integrative Genomics Viewer (IGV) was used to visualize Tcf7l2 peaks [49].

### 2.18 Immunoblotting analyses

Tissue samples were homogenized with a BioVortexer mixer (Chemglass, Vineland, NJ, USA) in ice-cold lysis buffer (1% SDS, 12.7 mM EDTA, 60 mM Tris–HCl, and pH 6.8) containing 1:100 protease inhibitor cocktail (Sigma–Aldrich, St. Louis, MO, USA). Lysates were centrifuged at 13,600×*g* for 10 min at 4 °C, the top lipid layer was removed, and extracts were centrifuged again. Cultured cells were washed twice with PBS, lysed in ice-cold lysis buffer containing 1:100 protease inhibitor cocktail, and homogenized. Lysates were then centrifuged at 13,600×*g* for 10 min at 4 °C. Protein concentrations of tissue or cell lysates were measured by BCA protein assays (Thermo Fisher Scientific, Waltham, MA, USA). Lysates were diluted to equal protein concentrations in Laemmli sample buffer and lysis buffer, vigorously vortexed, and denatured at 95 °C for 5 min. Tissue or cell extracts (20 μg) were separated by SDS-PAGE on 4–12% gradient polyacrylamide gels (Invitrogen, Carlsbad, CA, USA) and transferred to Immobilon PVDF membranes (Millipore, Billerica, MA, USA). Prior to immunoblotting with primary antibodies, membranes were blocked in 5% non-fat dried milk in Tris-buffered saline, pH 7.4, containing 0.05% Tween-20 (TTBS) for 1 h at room temperature. All primary antibodies were used at a concentration of 1:1000 in TTBS containing 5% BSA overnight at 4 °C. Membranes were probed with horseradish peroxidase-conjugated secondary antibodies (1:5000) diluted in 5% non-fat dried milk in TTBS for 1.5 h at room temperature and subsequently visualized with Clarity Western ECL Substrate (Bio-Rad, Hercules, CA, USA) or SuperSignal West Femto Maximum Sensitivity Substrate (Thermo Fisher Scientific, Waltham, MA, USA). Primary antibodies are included in Supplemental Table 2.

### 2.19 Statistics

All data are presented as mean ± S.D. Significance was determined using two-tailed Student's t-test when comparing two groups. An analysis of variance (ANOVA) was followed by post hoc analyses with Dunnett's or Sidak's test, as appropriate, when comparing multiple experimental groups. Observed differences were considered significant at p < 0.05 and are indicated with asterisks.

## 3 Results

### 3.1 β-catenin is highly expressed in terminally differentiated adipocytes and upregulated by obesity

**Figure 1:**
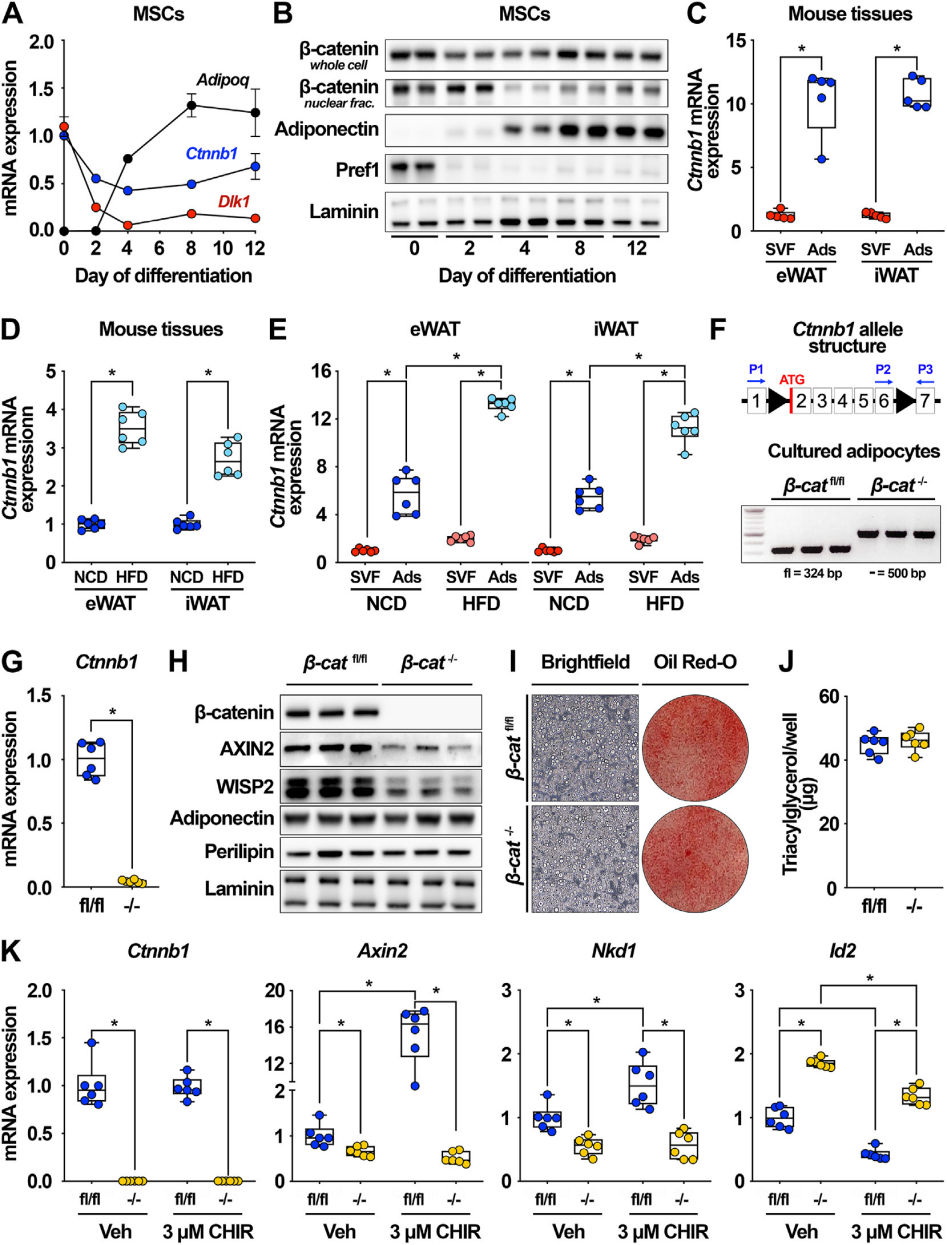
**β-catenin is expressed in cultured and primary adipocytes and up-regulated by diet-induced obesity.** (**A-B**) Mesenchymal stem cells (MSC) isolated from C57BL/6J mice were cultured under standard conditions and induced to differentiate. *Ctnnb1* gene (n = 6) and protein (n = 2) expression at indicated days of adipogenesis. (**C**) *Ctnnb1* gene expression in stromal-vascular (SVF) and adipocyte (Ads) fractions isolated from epididymal (eWAT) and inguinal (iWAT) white adipose tissues (WAT) of C57BL/6J mice (males; n = 5). (**D**) Expression of *Ctnnb1* in eWAT and iWAT of mice fed a normal chow diet (NCD) or high-fat diet (HFD) for 10 weeks. (**E**) *Ctnnb1* expression in SVF and Ads of eWAT and iWAT isolated from NCD- and HFD-fed mice (males; n = 6). (**F**) *Ctnnb1* allele structure and genetic recombination in *β-cat*^fl/fl^ and *β-cat*^−/−^ adipocytes using a 3-primer PCR system (n = 3). (**G-H**) *Ctnnb1* RNA (n = 6) and protein (n = 3) expression in adipocytes following adenoviral GFP or Cre infection. (**I**) Representative brightfield and Oil Red O images, and (**J**) triacylglycerol (TAG) accumulation in *β-cat*^fl/fl^ and *β-cat*^−/−^ adipocytes (n = 6). (**K**) Expression of *Ctnnb1* and downstream Wnt target genes in *β-cat*^fl/fl^ and *β-cat*^−/−^ adipocytes treated with vehicle or 3 μM CHIR99021 for 4 h (n = 6). RNA expression normalized to PPIA. Data presented as mean ± S.D. ∗ indicates significance at p < 0.05.

The canonical Wnt/β-catenin pathway is a critical regulator of mesenchymal cell fate determination and an endogenous inhibitor of adipogenesis [15,16]. Although Wnt signaling is commonly believed to be most important in precursors, recent studies have begun to shed light on contributions of this pathway to functions within terminally-differentiated adipocytes [[37], [38], [39], [40]]; however, specific roles of β-catenin in this context require further investigation. We thus first confirmed that β-catenin is expressed in mature adipocytes. *Ctnnb1* (β-catenin) mRNA and protein expression is high in cultured mesenchymal stem cells (MSC) derived from wild-type C57BL/6J mice, transiently suppressed during differentiation, and subsequently increased in terminally-differentiated adipocytes (Figure 1A–B). Expression of the adipocyte marker *Adipoq* (adiponectin) increases during adipogenesis, whereas expression of the preadipocyte marker *Dlk1* (Pref1) decreases as expected (Figure 1A–B).

To determine β-catenin levels in adipose tissues *in vivo*, we performed immunoblotting analyses of various tissues isolated from wild-type mice and found that β-catenin is widely expressed, including in diverse adipose depots, pancreas, and lung (Supplemental Figure 1A). In addition to adipocytes, WAT is comprised of many other cell types, including endothelial, immune, and stromal cells and preadipocytes. These non-adipocyte cells are known as the stromal-vascular fraction (SVF) of WAT, and previous studies have demonstrated that β-catenin is widely expressed in many of these populations [15,16,[50], [51], [52], [53]]. To assess whether adipocyte-specific expression contributes significantly to β-catenin levels within whole adipose tissues, we measured *Ctnnb1* mRNA expression in fractionated epididymal (eWAT) and inguinal (iWAT) WAT of wild-type mice. We found that *Ctnnb1* is highly expressed in isolated adipocytes compared to the SVF (Figure 1C), consistent with recently reported data [37]. As expected, *Adipoq* expression is found exclusively in the adipocyte fractions, whereas *Dlk1* expression is limited to the SVF (Supplemental Figure 1B), providing evidence of clean separation.

Recent studies have demonstrated that Wnt signaling plays a role in adipocyte metabolism under obesogenic conditions [[37], [38], [39], [40]]. Thus, we examined the regulation of β-catenin in WAT after a number of nutritional and environmental treatments and found that its expression is upregulated in both eWAT and iWAT with diet-induced obesity (Figure 1D), parallel with induction of *Lep* (leptin) (Supplemental Figure 1C). Consistent with *Ctnnb1* expression patterns, downstream Wnt target *Nkd1* increases in eWAT with HFD treatment; effects in iWAT are not statistically different (Supplemental Figure 1D). Further, analyses of fractionated WAT demonstrate that upregulation of *Ctnnb1* largely occurs in adipocytes, rather than in the SVF (Figure 1E). This regulation is specific; *Ctnnb1* expression is not altered by other conditions, including acute fasting, fasting/refeeding, calorie restriction, or acute cold exposure (Supplemental Figure 1E). Finally, we searched the GTEx database to determine the distribution of *Ctnnb1* gene expression across male and female human tissues and found that β-catenin is highly expressed in subcutaneous and visceral WAT depots, with relatively low expression in the liver, skeletal muscle, and pancreas (Supplemental Figure 1F). Together, these data demonstrate that β-catenin is expressed in mature adipocytes and up-regulated by diet-induced obesity, and thus conceivably plays an important metabolic role in these cells.

### 3.2 Canonical Wnt signaling is operative in cultured adipocytes

To assess the molecular functions of β-catenin in adipocytes, we used a cultured cell model using MSCs isolated directly from *β-cat*^fl/fl^ mice. To efficiently ablate β-catenin in either preadipocytes or adipocytes, we established a gene deletion protocol using adenoviral Cre recombinase (Supplemental Figure 1G). To induce gene deletion in preadipocytes, *β-cat*^fl/fl^ MSCs at 30–40% confluence were infected in serum-free medium with adenoviral GFP as a control or adenoviral Cre to induce recombination. Preadipocytes were allowed to recover following infection and were analyzed at confluence. To induce gene deletion in adipocytes, *β-cat*^fl/fl^ MSCs were differentiated using a standard adipogenic cocktail and on day four of differentiation, adipocytes were infected with adenoviral GFP or Cre in serum-free medium. The cells were then allowed to recover and were subsequently analyzed on day 12 of differentiation. A 3-primer PCR system was used to confirm recombination of the floxed allele (Figure 1F, preadipocytes shown in Supplemental Figure 1H). Efficient deletion of both *Ctnnb1* mRNA (Figure 1G) and protein (Figure 1H, preadipocytes shown in Supplemental Figure 1H) were observed in Cre-infected adipocytes. Adipocyte-specific β-catenin deletion did not affect the differentiation status or lipid accumulation, as evidenced by adiponectin and perilipin protein expression (Figure 1H), phase-contrast microscopy, Oil Red O staining (Figure 1I), and quantification of triacylglycerol (TAG) content per well (Figure 1J). Importantly, protein levels of AXIN2 and WISP2, two known Wnt targets, decreased in *β-cat*^−/−^ adipocytes (Figure 1H, preadipocytes shown in Supplemental Figure 1H).

Thus, we next investigated in more detail whether Wnt target genes are altered in adipocytes lacking β-catenin. To this end, we measured the expression of downstream genes under basal conditions and after stimulation with the small molecule CHIR99021, which stabilizes β-catenin through inhibition of GSK3 activity [15]. We found that known Wnt-induced genes, including *Axin2*, *Nkd1*, and *Tcf7l2*, were down-regulated in *β-cat*^−/−^ adipocytes, whereas Wnt-repressed genes *Id2* and *Wif1* were up-regulated (Figure 1K, Supplemental Figure 1I). Importantly, Wnt pathway stimulation with CHIR99021 induced expression of most target genes only in *β-cat*^fl/fl^ adipocytes and not in *β-cat*^−/−^ cells, suggesting that the expression of these genes is β-catenin-dependent in terminally-differentiated cells (Figure 1K, Supplemental Figure 1I). Of note, *Id2* expression was down-regulated in both control and knockout adipocytes with CHIR99021 treatment, albeit to different degrees, suggesting that it is also regulated by other cellular pathways affected by GSK3 inhibition (Figure 1K). Taken together, these data indicate that the canonical Wnt signaling pathway is operative in mature adipocytes and likely regulates functions that are specialized to these cells.

### 3.3 β-catenin regulates metabolic pathways in adipocytes and exclusively mediates effects of canonical Wnt3a signaling

**Figure 2:**
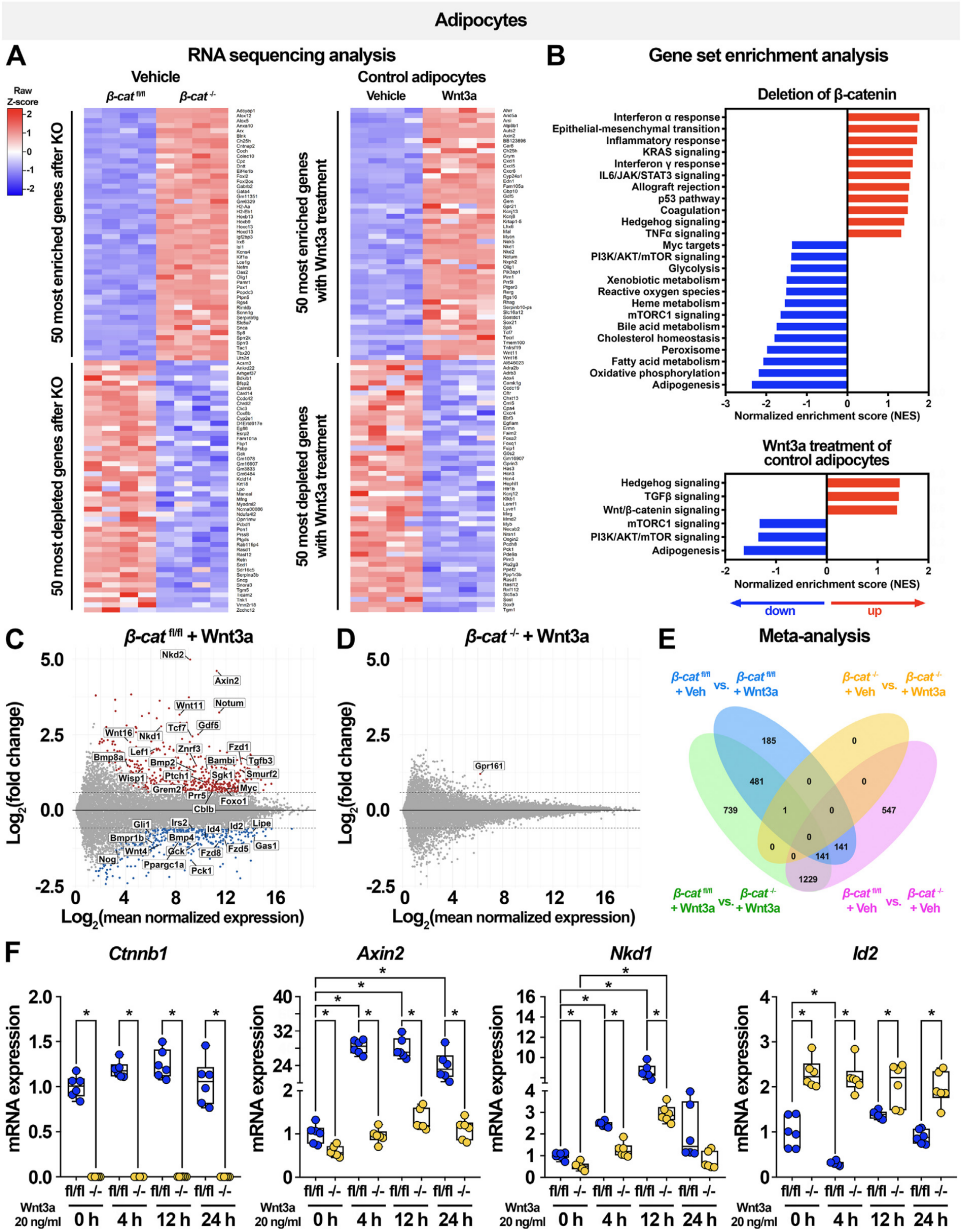
**β-catenin regulates metabolic pathways in adipocytes and exclusively mediates effects of canonical Wnt3a signaling.** RNA-seq analyses were performed on *β-cat*^fl/fl^ and *β-cat*^−/−^ adipocytes under basal conditions or after 4 h of treatment with recombinant Wnt3a (20 ng/ml; n = 4 per group). (**A**) Heat maps of differential gene expression changes in *β-cat*^fl/fl^ and *β-cat*^−/−^ adipocytes under basal conditions (left panel) and *β-cat*^fl/fl^ cells treated with vehicle or Wnt3a (right panel). (**B**) Gene Set Enrichment Analyses (GSEA) of genes expressed in *β-cat*^fl/fl^ and *β-cat*^−/−^ adipocytes under basal conditions (top panel) and *β-cat*^fl/fl^ cells treated with vehicle or Wnt3a (bottom panel). (**C-D**) MA plots of gene expression changes following Wnt3a treatment of *β-cat*^fl/fl^ or *β-cat*^−/−^ adipocytes. (**E**) Venn diagram depicting meta-analysis of gene expression changes in *β-cat*^fl/fl^ or *β-cat*^−/−^ adipocytes treated with vehicle or Wnt3a for 4 h. (**F**) Expression of *Ctnnb1* and downstream Wnt target genes in *β-cat*^fl/fl^ and *β-cat*^−/−^ adipocytes treated with vehicle or 20 ng/ml recombinant Wnt3a for 4, 12, or 24 h (n = 6). RNA expression normalized to PPIA. Data presented as mean ± S.D. ∗ indicates significance at p < 0.05.

Although β-catenin-dependent canonical Wnt signaling is a critical regulator of mesenchymal cell fate, the role of this pathway in mature adipocytes is less clear. To identify transcriptional pathways directly regulated by β-catenin in terminally-differentiated cells, we performed RNA sequencing (RNA-seq) analyses of *β-cat*^fl/fl^ and *β-cat*^−/−^ adipocytes at baseline and following 4 h of treatment with recombinant Wnt3a (Figure 2A,E). Of the 18,931 identified genes, β-catenin deletion in adipocytes resulted in significant up-regulation of 1,582 genes and down-regulation of 484 genes. We then used GSEA to identify significantly up- and down-regulated pathways in knockout cells. Strikingly, the majority of up-regulated pathways were related to the inflammatory response, including TNFα, IL6, interferon γ, and interferon α signaling; in contrast, loss of β-catenin led to the suppression of several metabolic pathways, including those involved in glycolysis, oxidative phosphorylation, and fatty acid, cholesterol, and bile acid metabolism (Figure 2B). Of note, mTORC1 signaling was also significantly down-regulated in *β-cat*^−/−^ adipocytes, which is of interest since this pathway has been shown to regulate lipid metabolism in adipocytes [[54], [55], [56], [57]].

Wnt3a treatment of *β-cat*^fl/fl^ adipocytes resulted in significant up-regulation of 535 genes and down-regulation of 417 genes, including dramatic induction of known Wnt targets *Axin2*, *Nkd1*, *Nkd2*, and *Wnt11* (Figure 2A,C). Consistent with canonical Wnt/β-catenin signaling predominating in adipocytes, Wnt3a treatment of *β-cat*^−/−^ adipocytes only altered expression of one gene, *Gpr161*, which has no known function (Figure 2D). As expected, treatment of control adipocytes with Wnt3a stimulated the expression of genes known to be related to Wnt/β-catenin signaling (Figure 2B). Hedgehog and TGFβ signaling, which have broad effects on differentiation and mature cell functions, were also up-regulated (Figure 2B); cross-talk between these pathways and Wnt signaling has been reported in other contexts [58,59]. Differential regulation of a subset of Wnt target genes was confirmed by qPCR analyses of control and knockout adipocytes treated with Wnt3a for different lengths of time (Figure 2F).

Consistent with extensive reports describing the role of Wnt signaling in precursors, RNA-seq analyses of *β-cat*^fl/fl^ and *β-cat*^−/−^ preadipocytes identified 866 up-regulated genes and 936 down-regulated genes (Supplemental Figure 2A and E). Deletion of β-catenin in precursors suppressed many pathways critical for cell function, including DNA repair, proliferation, stress response, and glycolysis (Supplemental Figure 2B), consistent with known roles of Wnt signaling in cell proliferation and cancer metabolism [60]. Similar to observations in control adipocytes, Wnt3a treatment of *β-cat*^fl/fl^ preadipocytes promoted the expression of many genes related to TGFβ, Hedgehog, and Notch signaling (Supplemental Figure 2A-C). Unlike in *β-cat*^−/−^ adipocytes, Wnt3a treatment of *β-cat*^−/−^ preadipocytes resulted in up regulation of 146 genes and down-regulation of 171 genes, including induction of Wnt target genes such as *Axin2* and *Nkd1* (Supplemental Figure 2D-F). These data indicate that in preadipocytes, Wnt target genes are also regulated by non-canonical Wnt signaling pathways, whereas in adipocytes, Wnt3a acts exclusively through a β-catenin-dependent mechanism.

### 3.4 β-catenin-dependent signaling regulates lipogenesis and fatty acid desaturation in adipocytes

**Figure 3:**
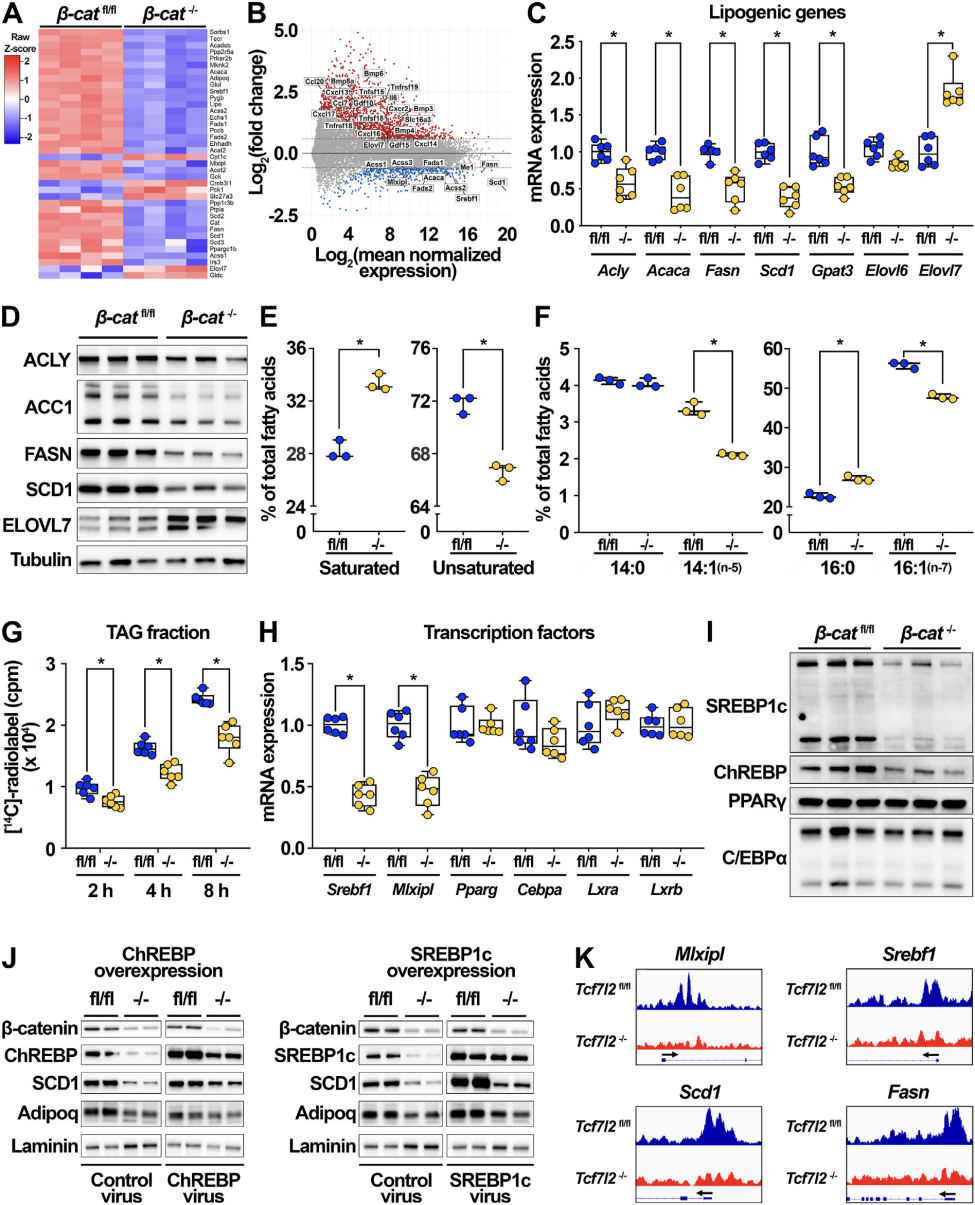
**β-catenin-dependent Wnt signaling regulates lipogenesis and fatty acid desaturation in adipocytes.** (**A-B**) Heat map and MA plot showing differentially expressed genes related to fatty acid, cholesterol, and bile acid metabolism in cultured *β-cat*^fl/fl^ and *β-cat*^−/−^ adipocytes (n = 4). (**C-D**) Lipogenic gene (n = 6) and protein (n = 3) expression in *β-cat*^fl/fl^ and *β-cat*^−/−^ adipocytes. (**E**) Proportion of total saturated vs unsaturated fatty acids in lipids extracted from *β-cat*^fl/fl^ and *β-cat*^−/−^ adipocytes (n = 3). (**F**) Relative proportions of myristic (C14:0) and palmitic (C16:0) vs myristoleic (C14:1, n-5) and palmitoleic (C16:1, n-7) acids (n = 3). (**G**) *De novo* lipogenesis (DNL) was evaluated in cultured *β-cat*^fl/fl^ and *β-cat*^−/−^ adipocytes using [^14^C]-acetate for 2, 4, and 8 h. Incorporation of [^14^C]-radiolabel into TAG fractions extracted from *β-cat*^fl/fl^ and *β-cat*^−/−^ adipocytes was quantified by scintillation counting (n = 6). (**H–I**) Gene (n = 6) and protein (n = 3) expression of indicated transcription factors in *β-cat*^fl/fl^ and *β-cat*^−/−^ adipocytes. (**J**) Protein expression in *β-cat*^fl/fl^ and *β-cat*^−/−^ adipocytes treated for 72 h with adenovirus expressing GFP, ChREBP, or SREBP1c (1 × 10^5^ viral particles/ml). (**K**) Integrative Genomics Viewer capture showing Tcf7l2 peaks (indicating binding occupancy) in regions ± 3 kb from transcription start sites (black arrows) of the indicated genes in cultured *Tcf7l2*^fl/fl^ and *Tcf7l2*^−/−^ adipocytes. RNA expression normalized to PPIA. Data presented as mean ± S.D. ∗ indicates significance at p < 0.05.

Lipogenesis and lipid accumulation are two specialized functions of adipocytes. Thus, we were intrigued to find that the RNA-seq dataset identified many genes related to lipid metabolism, including *Srebf1 and Scd1*, as significantly down-regulated in *β-cat*^−/−^ adipocytes (Figure 3A–B). Consistent with the RNA-seq data, we found that β-catenin deficiency in adipocytes down-regulated mRNA (Figure 3C) and protein (Figure 3D, quantification in Supplemental Figure 3A) expression of many genes in the *de novo* lipogenesis (DNL) pathway, including ATP citrate lyase (*Acly*, ACLY), acetyl-CoA carboxylase (*Acaca*, ACC1), fatty acid synthase (*Fasn*, FASN), and stearoyl-CoA desaturase 1 (*Scd1*, SCD1). DNL converts excess dietary amino acids and carbohydrates into fatty acids, a subset of which can be esterified into TAG and later mobilized to provide energy to adipocytes and other cells throughout the body [61,62]. SCD1, a member of the DNL pathway, is the rate-limiting adipocyte enzyme localized to the endoplasmic reticulum that catalyzes desaturation of palmitic (C16:0) and stearic (18:0) acids into palmitoleic (C16:1, n-7) and oleic (C18:1, n-9) acids, respectively [63,64]. Because SCD1 protein expression was suppressed by ∼55% in *β-cat*^−/−^ adipocytes (Figure 3D, Supplemental Figure 3A), we hypothesized that TAG isolated from these cells would have decreased unsaturated fatty acids and increased saturated fatty acids. Indeed, we found that *β-cat*^−/−^ adipocytes contained a smaller proportion of monounsaturated fatty acid species compared to control adipocytes (Figure 3E). Closer examination revealed that compared to *β-cat*^fl/fl^ adipocytes, lipids isolated from terminally differentiated *β-cat*^−/−^ cells contained significantly higher proportions of palmitic (16:0) and stearic (18:0) acids and less myristoleic (14:1, n-5) and palmitoleic (16:1, n-7) acids (Figure 3F, Supplemental Figure 3B). In addition, *β-cat*^−/−^ adipocytes also contained slightly elevated proportions of oleic (C18:1, n-9), vaccenic (C18:1, n-7), and arachidonic (C20:4) acids (Supplemental Figure 3B, data not shown), consistent with previously published studies of SCD1 inhibition in 3T3-L1 adipocytes [65]. Although it is surprising that *Elovl6* did not follow the expression pattern of other ChREBP- and SREBP1-regulated lipogenic genes, increased *Elovl7* has been observed in various contexts of repressed Wnt signaling [40,[66], [67], [68]]. The functional significance of elevated ELOVL7 mRNA and protein remains unclear because we did not observe increased proportions of very long chain saturated fatty acids in *β-cat*^−/−^ adipocytes (data not shown).

As β-catenin deletion downregulates a network of key DNL pathway members (Figure 3A–D), we performed DNL assays using [^14^C]-acetate to evaluate whether lipogenesis is functionally impaired in *β-cat*^−/−^ adipocytes. Differentiated adipocytes were incubated in medium containing [^14^C]-acetate for 2, 4, or 8 h. Cells were then lysed and lipids extracted to measure incorporation of radiolabel into TAG, diacylglycerol (DAG), and phospholipid (PL) fractions. Conditioned media (Supplemental Figure 3C) and whole cell lysate (Supplemental Figure 3D) analyses indicated that *β-cat*^−/−^ adipocytes took up less labeled acetate over time. Consistent with this, both *Slc16a1* (*Mct1*), an acetate transporter, and *Acss2*, the cytosolic enzyme that catalyzes activation of acetate for use in lipid synthesis, were suppressed in *β-cat*^−/−^ adipocytes (Supplemental Figure 3B, E). *β-cat*^−/−^ adipocytes incorporated significantly less radiolabel over time into the TAG and DAG fractions of cellular lipid (Figure 3G, Supplemental Figure 3F), but not the PL fraction (Supplemental Figure 3G). This is important, as it suggests that acetate uptake is not the rate-limiting factor for radiolabel incorporation into TAG; indeed, decreased acetate incorporation in lipid synthesis may actually feedback to suppress transporter expression. Further, linear regression analyses demonstrated that decreased incorporation into TAG was not proportional to decreased acetate uptake (Supplemental Figure 3H-I), strongly suggesting that suppressed expression of DNL enzymes, not genes related to acetate transport or activation, is rate-limiting for lipogenesis in *β-cat*^−/−^ adipocytes. Thus, impaired DNL is secondary to loss of β-catenin-dependent Wnt signaling. These data suggest that adipocyte β-catenin signaling is required for lipogenic gene expression, DNL, and steady-state lipid composition.

### 3.5 β-catenin exerts effects on lipogenesis through transcriptional regulation of Srebf1 and Mlxipl

Extensive liver studies have identified SREBP1c and ChREBP as key upstream transcriptional regulators of many genes involved in DNL, including *Acaca*, *Fasn*, and *Scd1* [69,70]. We recently demonstrated for the first time that Wnt signaling mediates adipocyte lipogenic gene expression through regulation of sterol regulatory element-binding protein 1c (SREBP1c) and carbohydrate-responsive element-binding protein (ChREBP) [40]. Thus, we next investigated whether these genes are specifically regulated by β-catenin-dependent Wnt signaling. Indeed, we found that the expression of *Srebf1* and *Mlxipl*, which encode SREBP1c and ChREBP, respectively, were both repressed in *β-cat*^−/−^ adipocytes; other transcription factors involved in adipogenesis and mature adipocyte function, including *Pparg* and *Cebpa*, were not altered (Figure 3A–B, H). Consistent with expression of their respective mRNAs, SREBP1c and ChREBP protein levels were also decreased, whereas PPARγ and C/EBPα protein levels were unaffected (Figure 3I).

To establish whether repression of *Mlxipl* or *Srebf1* directly mediates impaired lipogenesis and fatty acid monounsaturation following β-catenin deletion, we evaluated the effects of increasing ChREBP or SREBP1c expression in β-catenin knockout adipocytes slightly above that observed in control adipocytes. Thus, we treated *β-cat*^fl/fl^ and *β-cat*^−/−^ adipocytes with adenoviral GFP as a control or adenoviruses encoding ChREBP or SREBP1c. Ectopic expression of either ChREBP or SREBP1c was sufficient to induce SCD1 expression in control adipocytes and partially rescue SCD1 mRNA and protein levels in *β-cat*^−/−^ adipocytes (Figure 3J, Supplemental Figure 3J). *Fasn* expression was also partially rescued by overexpression of these transcription factors (Supplemental Figure 3J).

As β-catenin coactivates TCF/LEF proteins such as Tcf7l2 (Tcf4) to mediate transcription of downstream targets, we analyzed the promoter regions of *Mlxipl*, *Srebf1*, *Acaca*, *Fasn*, and *Scd1* and found multiple predicted Tcf7l2 binding sites on each gene (data not shown). Thus, we interrogated a recently published chromatin immunoprecipitation sequencing (ChIP-seq) dataset of Tcf7l2 binding in cultured *Tcf7l2*^fl/fl^ and *Tcf7l2*^−/−^ adipocytes [39] and found enrichment of Tcf7l2 binding sites in the promoter, exon, and/or first intronic regions of *Mlxipl*, *Srebf1*, *Fasn*, and *Scd1*; binding occupancy of Tcf7l2 was subsequently lost in the *Tcf7l2*^−/−^ cells (Figure 3K). Taken together, these results support a model in which repressive effects of β-catenin deletion on lipogenic gene expression are mediated, in part, through down-regulation of ChREBP and SREBP1c. However, identification of Tcf7l2 binding sites in transcriptional regulatory regions of several lipogenic genes, including *Acaca*, *Fasn*, and *Scd1*, suggests that β-catenin regulates transcription of these genes directly through co-activation of TCF/LEF transcription factors and indirectly through expression of the lipogenic transcription factors ChREBP and SREBP1c.

### 3.6 Loss of β-catenin signaling within adipocytes does not influence global metabolism in chow-fed mice

**Figure 4:**
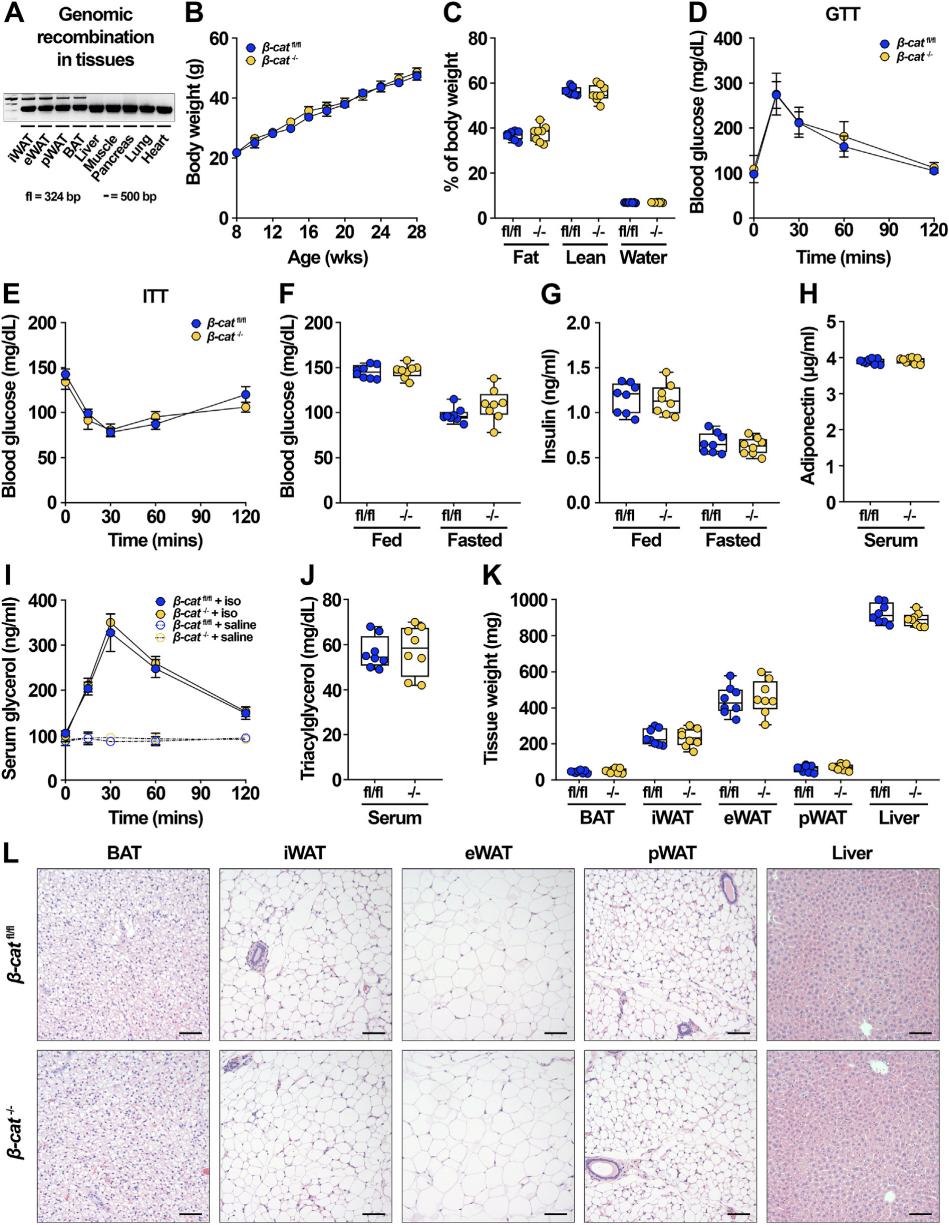
**Adipocyte-specific β-catenin deletion does not influence global metabolism on a normal chow diet.** (**A**) Genetic recombination in tissues isolated from *β-cat*^−/−^ mice. (**B**) Growth curves of 28-week-old *β-cat*^fl/fl^ and *β-cat*^−/−^ mice. (**C**) Body composition of 16-week-old *β-cat*^fl/fl^ and *β-cat*^−/−^ mice on NCD. (**D**) Glucose tolerance test in 16-week-old *β-cat*^fl/fl^ and *β-cat*^−/−^ mice. (**E**) Insulin tolerance test in 19-week-old mice. (**F**) Blood glucose concentrations in random-fed and 16 h fasted mice. Serum concentrations of (**G**) random-fed and fasted insulin and (**H**) adiponectin levels in 28-week-old mice. (**I**) Basal and stimulated lipolysis in 22-week-old mice (iso, isoproterenol: 10 mg/kg body weight). (**J**) Serum TAG in 28-week-old mice. (**K**) Tissue weights at time of sacrifice. (**L**) Representative histological images of H&E-stained tissues from *β-cat*^fl/fl^ and *β-cat*^−/−^ mice fed NCD for 28 weeks; 200x magnification; scale bar, 100 μm. Data in **B-L** from male mice, n = 8 per group. Data presented as mean ± S.D. ∗ indicates significance at p < 0.05.

Our studies in cultured adipocytes revealed that canonical Wnt signaling through β-catenin is an important regulator of adipocyte lipogenesis and lipid desaturation (Figure 3, Supplemental Figure 3). We next hypothesized that β-catenin has effects on these pathways *in vivo* and thus generated adipocyte-specific β-catenin knockout mice by crossing *β-cat*^fl/fl^ mice with adiponectin-Cre mice. Genetic recombination was specifically observed in various adipose depots, but not in other tissues, including the liver, muscle, pancreas, lung, or heart (Figure 4A). We evaluated possible metabolic phenotypes in *β-cat*^−/−^ mice maintained on a normal chow diet (NCD) but did not observe differences in growth over time (Figure 4B), body composition (Figure 4C), or glucose and insulin tolerance (Figure 4D–E). *β-cat*^−/−^ mice did not exhibit altered fed or fasted blood glucose (Figure 4F), serum insulin concentrations (4G), or circulating adiponectin levels (Figure 4H). We did not detect differences in voluntary exercise capacity (Supplemental Figure 4A), basal or induced lipolysis (Figure 4I), or serum TAG levels (Figure 4J). Upon harvest, weights of iWAT, eWAT, brown adipose tissue (BAT), perirenal WAT (pWAT), and liver were not different between *β-cat*^fl/fl^ and *β-cat*^−/−^ mice (Figure 4K). Histological analyses of tissues did not yield substantial differences in adipocyte size or number within BAT, iWAT, eWAT, or pWAT (Figure 4L, data not shown), and liver morphology was not influenced (Figure 4L). Similar results were found in female mice (Supplemental Figure 4B-J).

Wnt signaling through β-catenin has been shown to have profound effects on determination of MSC fate and differentiation into adipocytes, osteocytes, chondrocytes, or myocytes. The role of this pathway in fate selection between adipogenesis and osteogenesis has been particularly well-researched. Indeed, studies have demonstrated that Wnt signaling increases bone mass and trabeculation and impairs the accumulation of bone marrow adipocytes [71,72]. We therefore examined whether marrow adiposity and bone characteristics are influenced by adipocyte-specific β-catenin deficiency. Histological analyses of tibias from both male and female mice suggested that β-catenin deletion did not alter the size or number of regulated or constitutive marrow adipocytes (Supplemental Figure 5A and C). Further, micro-computed tomography (μCT) analyses did not yield differences in either trabecular or cortical bone mass variables in *β-cat*^−/−^ mice (Supplemental Figure 5B and D).

### 3.7 β-catenin is up-regulated in the stromal-vascular fraction of adipose tissues from knockout mice

**Figure 5:**
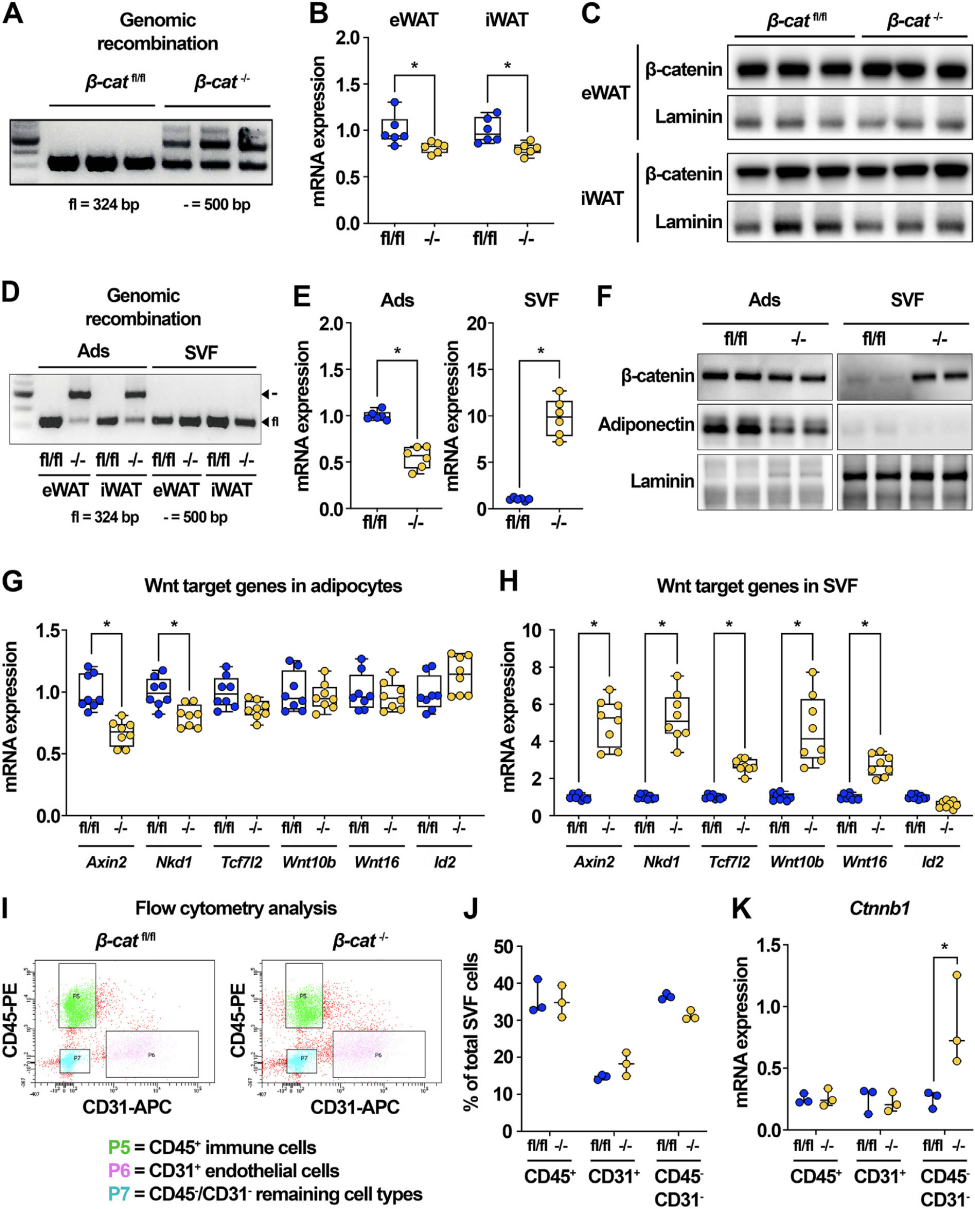
**β-catenin is up-regulated in the stromal-vascular fraction of adipose tissues from knockout mice.** (**A**) Genetic recombination in tissues isolated from *β-cat*^fl/fl^ and *β-cat*^−/−^ mice (n = 3). (**B–C**) *Ctnnb1* mRNA (n = 3) and protein (n = 6) expression in eWAT and iWAT of *β-cat*^fl/fl^ and *β-cat*^−/−^ mice. (**D**) Genomic recombination of β-catenin in adipocytes and SVF isolated from eWAT and iWAT of *β-cat*^fl/fl^ and *β-cat*^−/−^ mice. (**E**) *Ctnnb1* mRNA expression in isolated eWAT adipocytes and SVF of *β-cat*^fl/fl^ and *β-cat*^−/−^ mice (n = 6). (**F**) β-catenin protein expression in isolated eWAT adipocytes and SVF of *β-cat*^fl/fl^ and *β-cat*^−/−^ mice; adiponectin and laminin shown as protein loading controls. (**G-H**) Wnt target gene expression in adipocytes and SVF isolated from eWAT of *β-cat*^fl/fl^ and *β-cat*^−/−^ mice (n = 8). (**I**) Representative plots showing flow cytometry analysis of SVF isolated from *β-cat*^fl/fl^ and *β-cat*^−/−^ mice (3 mice per sample; n = 3 samples). (**J**) Quantification of SVF cell proportions evaluated by flow cytometry analysis (3 mice per sample; n = 3 samples). (**K**) *Ctnnb1* mRNA expression normalized to PPIA in cellular fractions isolated by FACS analysis (3 mice per sample; n = 3 samples). Data presented as mean ± S.D. ∗ indicates significance at p < 0.05.

Although we did not observe an overt metabolic phenotype in *β-cat*^−/−^ mice maintained on chow diets, we expected to find decreased DNL gene expression in eWAT and iWAT isolated from knockout mice. However, lipogenic gene expression was not influenced in whole eWAT or iWAT of *β-cat*^−/−^ mice (Supplemental Figure 6A-B). We also did not observe changes in DNL gene expression within livers of the *β-cat*^−/−^ mice (Supplemental Figure 6C). Perhaps most perplexing, analyses of isolated adipocytes from *β-cat*^−/−^ mice did not demonstrate altered DNL gene expression, except for mild suppression of *Scd1* (Supplemental Figure 6D). These data were concerning and suggested that perhaps β-catenin was not efficiently deleted *in vivo*. Indeed, we were surprised to find that although β-catenin appeared to be recombined at the genomic level in whole WAT of *β-cat*^−/−^ mice (Figure 5A), suppression of *Ctnnb1* mRNA was much less substantial than expected (Figure 5B). Further, β-catenin protein levels were virtually unaltered in eWAT and iWAT of *β-cat*^−/−^ mice (Figure 5C).

As SVF cell populations are known to express β-catenin, we considered the possibility that adipocyte-specific loss was being masked by the relatively high expression of β-catenin in SVCs. Thus, we fractionated WAT to determine whether efficient deletion occurred within adipocytes. Indeed, we found that β-catenin gene recombination occurred specifically in adipocytes and not SVCs of *β-cat*^−/−^ mice (Figure 5D). However, we were surprised to find that *Ctnnb1* mRNA was reduced by only ∼50% in the adipocyte fraction of *β-cat*^−/−^ mice, and its expression was highly induced in the SVF of knockout mice (Figure 5E). We also observed sustained levels of β-catenin protein in knockout adipocytes and significant induction of β-catenin protein within the SVF of *β-cat*^−/−^ mice (Figure 5F). We next measured Wnt target genes in isolated adipocytes and SVCs of *β-cat*^fl/fl^ and *β-cat*^−/−^ mice. Consistent with β-catenin expression patterns, we found that most Wnt targets were not altered in the adipocyte fraction of *β-cat*^−/−^ mice (Figure 5G), whereas these genes were significantly up-regulated in the SVF with β-catenin deficiency (Figure 5H).

These striking findings suggest that adipose tissues can sense depletion of β-catenin and subsequently maintain canonical Wnt signaling across the tissue by up-regulating the pathway in SVCs. Thus, we next evaluated whether a specific cellular sub-population was enriched within the SVF of *β-cat*^−/−^ mice and found that several macrophage markers, including *Adgre1* (*F4/80*), *Cd68*, and *Cd11c*, were elevated, whereas markers for endothelial and stromal cells did not change (Supplemental Figure 6E). These data may suggest that macrophage numbers were increased in the SVF of *β-cat*^−/−^ mice; these macrophages may in turn have contributed *Ctnnb1* mRNA or β-catenin protein back to deficient adipocytes, either directly via small extracellular vesicles (sEVs) or indirectly by stimulating production in neighboring cells. Although macrophage markers were increased, expression of classical inflammatory markers were either decreased or unchanged in whole WAT of *β-cat*^−/−^ mice (Supplemental Figure 6F).

We next performed flow cytometry analysis of the SVF isolated from chow-fed *β-cat*^fl/fl^ and *β-cat*^−/−^ mice. We used CD45 and CD31 as markers for immune and endothelial cells, respectively; CD45^-^/CD31^-^ cells were designated as the stromal cell population. Flow cytometry analysis did not yield differences in proportions of the CD45^+^, CD31^+^, or CD45^-^/CD31^-^ populations (Figure 5I–J). Since proportions of different SVF cell types were not influenced by adipocyte-specific β-catenin deletion, we next hypothesized that a specific cell type might have up-regulated its own β-catenin expression. Thus, we used fluorescence-activated cell sorting (FACS) to separate CD45^+^, CD31^+^, and CD45^-^/CD31^-^ cell fractions. The expression of *F4/80*, *Pecam1*, and *Pdgfra* was evaluated by qPCR to confirm that we had specifically separated immune, endothelial, and stromal cell populations (Supplemental Figure 6G). We next measured *Ctnnb1* mRNA expression in the three cell fractions and were intrigued to find that *Ctnnb1* was up-regulated in the CD45^-^/CD31^-^ population of chow-fed *β-cat*^−/−^ mice (Figure 5K). Consistent with *Ctnnb1* expression patterns, downstream Wnt targets *Axin2* and *Nkd1* were also increased in the CD45^-^/CD31^-^ cells isolated from *β-cat*^−/−^ mice (Supplemental Figure 6H). These data suggest that a sub-population of CD45^-^/CD31^-^ stromal cells is able to sense the loss of adipocyte β-catenin, either directly or indirectly, and subsequently up-regulate its own expression to maintain Wnt signaling homeostasis within WAT of chow-fed mice. Further studies will be required to identify the specific cells within this population that mediate the observed compensatory effects.

### 3.8 *β-cat*^−/−^ mice are protected from diet-induced obesity and metabolic dysfunction

**Figure 6:**
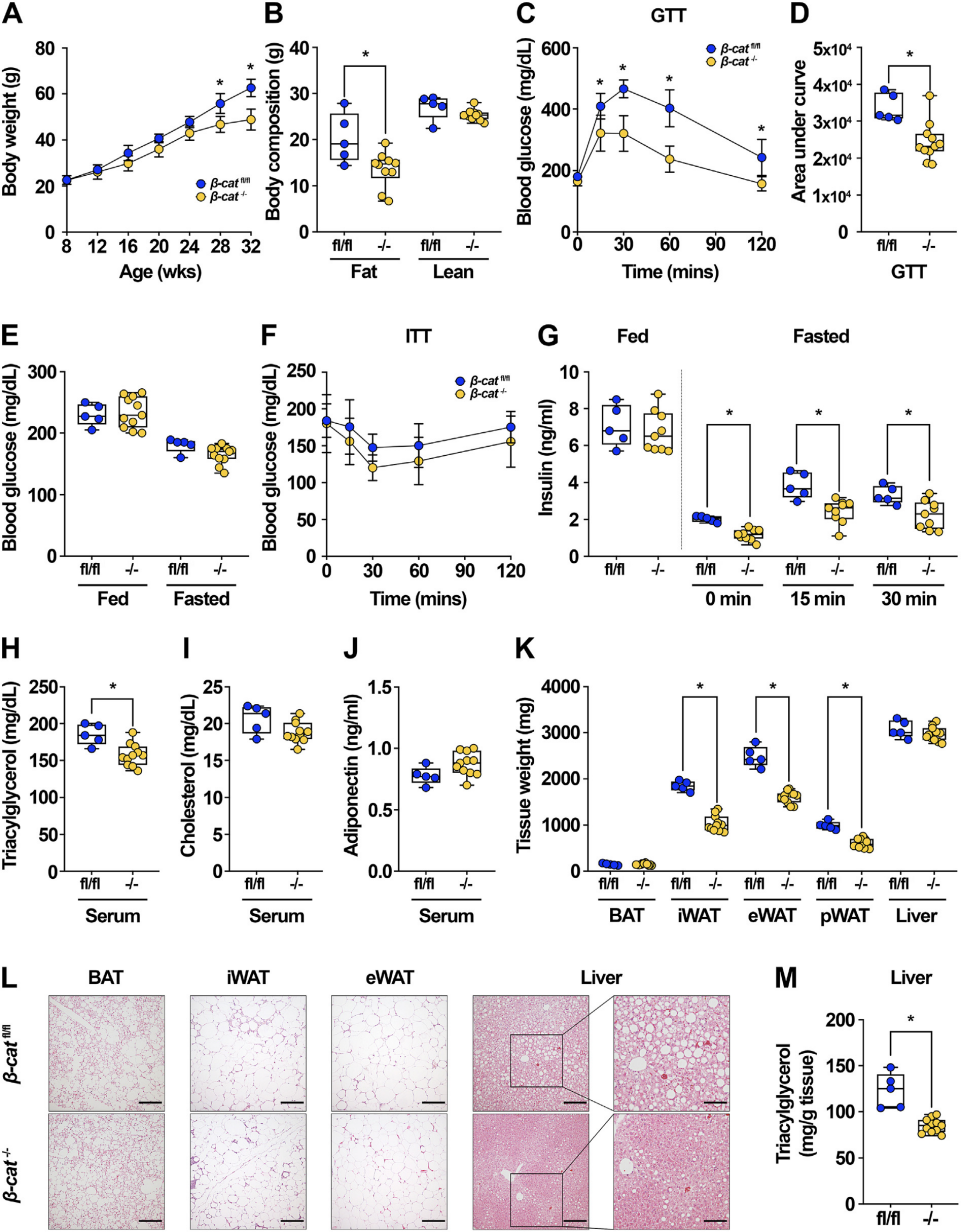
***β-cat***^**−/−**^**mice are protected from diet-induced obesity and metabolic dysfunction.** (**A**) Growth curves over time of 32-week-old *β-cat*^fl/fl^ and *β-cat*^−/−^ mice fed 60% HFD for 24 weeks. (**B**) Body composition analysis of 28-week-old mice. (**C-D**) Glucose tolerance test and area under the curve analysis in 28-week-old mice. (**E**) Blood glucose concentrations in random-fed and 16 h fasted mice. (**F**) Insulin tolerance test in 30-week-old mice. (**G**) Serum insulin concentrations in random-fed mice or 16 h fasted mice at indicated times after intraperitoneal glucose injection (1 mg/kg body weight). Serum (**H**) TAG, (**I**) total cholesterol, and (**J**) adiponectin in 32-week-old mice. (**K**) Tissue weights at time of sacrifice. (**L**) Representative histological images of H&E-stained tissues from *β-cat*^fl/fl^ and *β-cat*^−/−^ mice fed HFD for 24 weeks; 200x magnification; scale bar, 100 μm. (**M**) Quantification of liver TAG in 32-week-old mice. Data shown from male mice; *β-cat*^fl/fl^: n = 5, *β-cat*^−/−^: n = 11. Data presented as mean ± S.D. ∗ indicates significance at p < 0.05.

Previous investigations into the role of Wnt signaling in WAT have found that this pathway is important in adipocyte metabolism under obesogenic conditions [[37], [38], [39], [40]]. Consistent with these studies, we report that *Ctnnb1* expression was up-regulated within both eWAT and iWAT with diet-induced obesity (Figure 1D–E). We thus challenged *β-cat*^fl/fl^ and *β-cat*^−/−^ mice with HFD and found that beginning at 20 weeks of feeding, knockout mice demonstrated decreased weight gain (Figure 6A) and fat mass (Figure 6B) compared to control mice, whereas lean mass was not affected. Of note, *β-cat*^−/−^ mice did not have decreased food intake (Supplemental Figure 7A). Consistent with decreased adiposity, *β-cat*^−/−^ mice exhibited significantly improved glucose tolerance (Figure 6C–D). Although fasting and random-fed blood glucose concentrations were not different (Figure 6E) and insulin sensitivity only trended toward improvement in knockout mice (Figure 6F, Supplemental Figure 7B), glucose-induced circulating insulin concentrations were decreased in *β-cat*^−/−^ mice compared to controls (Figure 6G). Circulating TAG levels were decreased in knockout mice (Figure 6H), whereas serum cholesterol (Figure 6I) and adiponectin (Figure 6J) did not change. Upon harvest, weights of iWAT, eWAT, and pWAT were significantly decreased in knockout mice, consistent with leaner body composition, whereas BAT, liver, and pancreas weights were unchanged (Figure 6K, Supplemental Figure 7C). Histological analyses of *β-cat*^−/−^ tissues suggested that adipocyte sizes within iWAT and eWAT were mildly decreased (Figure 6L). In addition, livers of knockout mice had less hepatosteatosis compared to control counterparts (Figure 6L), in line with recently reported findings [37]. Consistent with the observed protection from glucose intolerance and the histological findings, livers of *β-cat*^−/−^ mice had decreased TAG content (Figure 6M). *Cdf* (adipsin) mRNA levels were increased in eWAT of *β-cat*^−/−^ mice, whereas *Lep* expression was decreased and *Adipoq* and *Retn* (resistin) remained unchanged; similar trends were observed in iWAT of knockout mice (Supplemental Figure 7D-E). The expression of UCP1 protein in BAT of *β-cat*^−/−^ mice was unchanged despite a ∼50% decrease in *Ctnnb1* expression (Supplemental Figure 7F); these data, along with comparable BAT morphology (Figure 6L), suggest that altered BAT thermogenesis was likely not responsible for improved metabolic function observed in obese β-catenin knockout mice.

**Figure 7:**
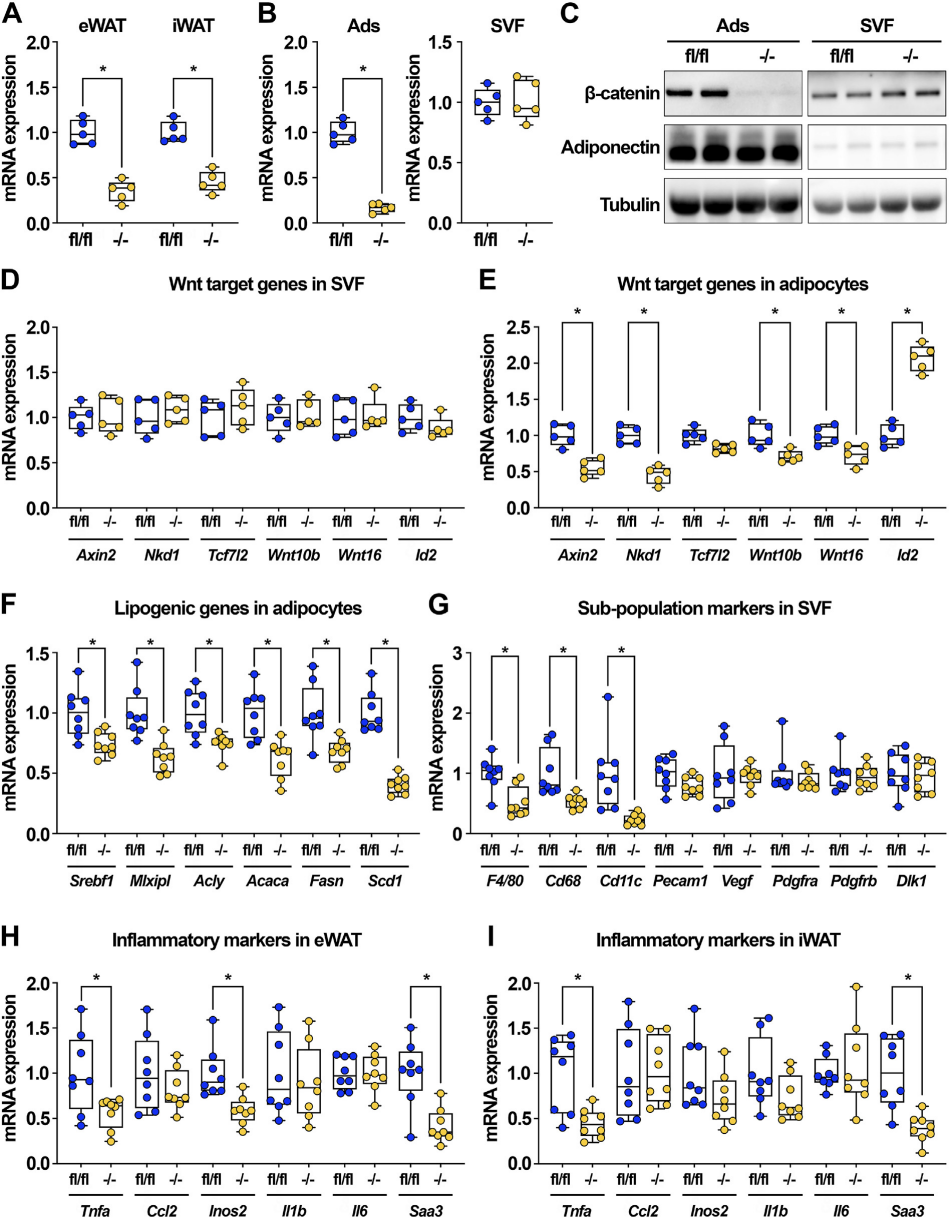
**Diet-induced obesity overcomes compensatory up-regulation of Wnt/β-catenin signaling in the SVF of knockout mice.** (**A**) *Ctnnb1* mRNA expression in eWAT and iWAT of *β-cat*^fl/fl^ and *β-cat*^−/−^ mice fed HFD for 28 weeks (n = 5). (**B**) *Ctnnb1* mRNA expression in isolated eWAT adipocytes and SVF of HFD-fed *β-cat*^fl/fl^ and *β-cat*^−/−^ mice (n = 5). (**C**) β-catenin protein expression in isolated eWAT adipocytes and SVF of *β-cat*^fl/fl^ and *β-cat*^−/−^ mice fed HFD; adiponectin and laminin shown as controls. (**D-E**) Wnt target gene expression in SVF and adipocytes isolated from eWAT of obese *β-cat*^fl/fl^ and *β-cat*^−/−^ mice (n = 5). (**F**) Lipogenic gene expression in eWAT adipocytes isolated from HFD-fed *β-cat*^fl/fl^ and *β-cat*^−/−^ mice (n = 8). (**G**) Expression of immune, endothelial, and stromal cell markers in SVF isolated from eWAT of obese *β-cat*^fl/fl^ and *β-cat*^−/−^ mice (n = 8). (**H–I**) Expression of inflammatory markers in whole eWAT and iWAT of *β-cat*^fl/fl^ and *β-cat*^−/−^ mice fed HFD (n = 8). RNA expression normalized to PPIA. Data presented as mean ± S.D. ∗ indicates significance at p < 0.05.

We next measured *Ctnnb1* expression in eWAT and iWAT of *β-cat*^−/−^ mice fed a HFD and found much lower mRNA levels in the tissues of knockout mice (Figure 7A). Further investigation revealed that *Ctnnb1* mRNA expression in isolated adipocytes was largely ablated in knockout mice, whereas the expression in SVF was comparable to that of control mice (Figure 7B). Consistent with these data, β-catenin protein was effectively deleted in the adipocyte fraction isolated from *β-cat*^−/−^ mice, and protein levels were no longer elevated in the SVF of these animals (Figure 7C). These data are compelling and suggest that with HFD, the compensatory increase in canonical Wnt signaling within stromal cells of knockout mice was lost. Indeed, the SVF of *β-cat*^−/−^ mice no longer exhibited elevated Wnt target gene expression (Figure 7D). Further, analysis of the adipocyte fraction demonstrated decreased expression of Wnt targets, including *Axin2*, *Nkd1*, *Wnt10b*, and *Wnt16* (Figure 7E).

Consistent with our *in vitro* studies showing the effects of β-catenin deletion on DNL, adipocytes of HFD-fed knockout mice were also characterized by decreased expression of key lipogenic genes, including *Srebf1*, *Mlxipl*, *Acly*, *Acaca*, *Fasn*, and *Scd1* (Figure 7F). We also found that macrophage markers were decreased in the SVF of HFD-fed knockout mice (Figure 7G), corroborating results reported by Chen et al. [37] and suggesting that these cells mediate the compensatory mechanism observed in chow-fed mice. Consistent with decreased immune cell markers, whole WAT of *β-cat*^−/−^ mice had suppressed expression of some inflammatory markers, including *Tnfa* and *Inos2* (Figure 7H–I). Expression of *Saa3*, which encodes a secreted protein that activates macrophages, was also decreased in WAT of HFD-fed knockout mice (Figure 7H–I). Thus, taken together, our findings demonstrate that diet-induced obesity overrides stromal compensation for adipocyte-specific β-catenin deletion, leading to impaired lipogenic gene expression, decreased adipose accumulation and adipocyte hypertrophy, and protection from metabolic dysfunction.

## 4 Discussion

Canonical β-catenin-dependent Wnt signaling is well-established as a key player in MSC fate determination, acting as a potent endogenous repressor of adipogenesis and promoter of osteoblastogenesis [10,12,13,15]. The preponderance of data within adipocyte biology to date has focused on the inhibition of adipogenesis by Wnt signaling, undoubtedly because many genes involved in this pathway are suppressed during the early stages of differentiation [73]. However, our work herein and recently published studies from our laboratory and others have demonstrated that canonical Wnt pathway members are present and operative in terminally-differentiated adipocytes and thus have distinct functional roles in this context [[37], [38], [39], [40]].

To evaluate specific roles of canonical Wnt signaling in adipocyte function, we ablated β-catenin, the central protein in the pathway. β-catenin deletion in cultured adipocytes suppressed expression of known downstream target genes, including *Axin2*, *Nkd1*, and *Tcf7l2*. Further, stimulation of Wnt signaling using either a GSK3 inhibitor or recombinant Wnt3a was largely blocked in adipocytes lacking β-catenin, suggesting that effects of canonical Wnt signaling are mediated exclusively by β-catenin in terminally-differentiated cells. Global RNA-seq analyses of *β-cat*^fl/fl^ and *β-cat*^−/−^ adipocytes identified several metabolic pathways as down-regulated by inhibition of Wnt signaling, including oxidative phosphorylation, glycolysis, and fatty acid, cholesterol, and bile acid metabolism. Of particular interest to adipocyte biology, we found that signaling through β-catenin is required for the coordinate expression of many lipogenic genes, including *Acly*, *Acaca*, *Fasn*, and *Scd1.* Of note, RNA-seq analyses of livers from *β-cat*^−/−^ mice also demonstrate decreased expression of lipogenic genes, including *Acly* and *Scd1* [74].

Consistent with repressed expression of DNL enzymes, *β-cat*^−/−^ adipocytes were characterized by impaired lipogenesis and fatty acid monounsaturation. Effects of β-catenin deletion on adipocyte metabolism were specific, as knockout adipocytes did not exhibit altered insulin-stimulated glucose uptake, adrenergic stimulation of lipolysis, or β-oxidation of fatty acids (data not shown). These data are strongly supported by recently published work from our group showing that blocking secretion and downstream signaling of adipocyte-derived Wnts also inhibits DNL and lipid unsaturation [40]. Additionally, Geoghegan et al. recently reported that *Tcf7l2* deletion in precursor cells stimulates adipogenesis and increased expression of genes related to lipid metabolism, providing further support for the presumptive role of β-catenin-dependent signaling in regulation of this process in adipocytes [39].

Extensive studies in the liver have identified *Srebf1* and *Mlxipl*, encoding SREBP1c and ChREBP, respectively, as key upstream transcriptional regulators of DNL enzymes [62]. In this study, we report that the expression of *Srebf1* and *Mlxipl* was significantly decreased in *β-cat*^−/−^ adipocytes, and that ectopic expression of ChREBP or SREBP1c partially rescued expression of DNL genes in adipocytes lacking β-catenin. These data are consistent with genome-wide ChIP-seq analysis of ChREBP binding sites in WAT, which identified binding sites on many genes related to metabolism, including *Acaca*, *Fasn*, and *Scd1* [75]. Thus, β-catenin mediates effects on DNL genes in part by regulating the expression of key transcription factors *Srebf1* and *Mlxipl*. Indeed, ChIP-seq analyses of Tcf7l2 binding sites in cultured adipocytes, used as a surrogate for β-catenin/TCF/LEF activity, identified specific Tcf7l2 occupancies in regions surrounding the transcriptional start sites of *Mlxipl* and *Srebf1*, but also downstream lipogenic genes such as *Fasn* and *Scd1*. Although these data suggest that β-catenin regulates transcription of lipogenic genes through a combination of indirect (via ChREBP and SREBP1c) and direct mechanisms, ChIP-seq analyses of β-catenin binding sites in adipocytes will be required to further elucidate the direct versus indirect effects of β-catenin on transcription of lipogenic genes.

Adipocyte-specific β-catenin deletion does not appear to influence global metabolism in chow-fed mice. Indeed, consistent with our studies, recent investigations into roles of Wnt signaling in mature adipocytes, including global *Sfrp5* deletion or adipocyte-specific deletion of *Tcf7l2*, *Wntless*, or *Ctnnb1*, also did not reveal an overt metabolic phenotype in chow-fed mice [[37], [38], [39], [40]]. However, we recently published a study showing that surrounding SVCs compensate for the loss of adipocyte-derived Wnts secondary to *Wntless* deletion [40]. Thus, we probed further into the lack of a detectable phenotype in *β-cat*^−/−^ mice and were surprised to find that although β-catenin was efficiently ablated at the genomic level in knockout adipocytes, the mRNA and protein were still detectable at much higher levels than expected. These data are consistent with those recently published by Chen et al., who reported a ∼50% reduction in *β-catenin* mRNA expression in adipocytes isolated from iWAT or eWAT of knockout mice [37]. Perhaps unsurprisingly, given the substantial β-catenin expression remaining, we found that Wnt targets and lipogenic genes were not altered in isolated knockout adipocytes. However, further investigation revealed that SVCs isolated from *β-cat*^−/−^ mice exhibited significantly up-regulated expression of *Ctnnb1* and downstream Wnt targets, including *Axin2*, *Nkd1*, *Tcf7l2*, *Wnt10b*, and *Wnt16*. Although Chen et al. did not report elevated *Ctnnb1* expression in the SVF of knockout mice, their data suggest a trend toward increased expression in SVF isolated from eWAT [37]. These data support the compelling conclusion that Wnt signaling is critical for autocrine and paracrine communication within WAT, such that loss of Wnt/β-catenin signaling in adipocytes is sensed and compensated for by SVCs to maintain whole-tissue Wnt signaling homeostasis.

An important question raised by our studies is the underlying mechanism by which β-catenin expression is up-regulated in SVF of knockout animals. Gene expression analysis of CD45^+^, CD31^+^, and CD45^-^/CD31^-^ populations isolated by FACS demonstrated that *Ctnnb1* and downstream targets *Axin2* and *Nkd1* are significantly upregulated in CD45^-^/CD31^-^ stromal cells of knockout mice. These data suggest that a sub-population of CD45^-^/CD31^-^ cells, which include adipose stem cells, committed preadipocytes, and pericytes [76], is able to directly or indirectly sense the loss of adipocyte β-catenin and subsequently up-regulates its own expression to maintain Wnt signaling homeostasis within WAT of chow-fed mice. This compensatory mechanism may be the result of a dynamic network of intercellular Wnt signals; alternatively, it may conceivably arise from complex interactions between canonical Wnt signaling and other pathways, including Hedgehog, BMP, and FGF signaling [58,77,78].

Gene expression analysis of whole SVF indicated that several macrophage markers, including *F4/80*, *Cd68*, and *Cd11c*, were increased in chow-fed *β-cat*^−/−^ mice. Of note, various isoforms of CD45 are present on almost all differentiated hematopoietic cells, and although *Cd68* and *Cd11c* are commonly used as macrophage markers, they are also expressed by non-hematopoietic cell types, including endothelial and stromal cells [79,80]. Thus, it is possible that β-catenin indeed causes a mild increase in macrophage number, but that this difference is not observed when using a broad marker such as CD45, which stains virtually all hematopoietic cells. Further studies using single-cell RNA sequencing analysis of SVF from *β-cat*^fl/fl^ and *β-cat*^−/−^ mice may help clarify specific sub-population changes following adipocyte-specific loss of β-catenin.

The relatively high levels of *Ctnnb1* mRNA and β-catenin protein in knockout adipocytes also suggests the intriguing possibility that β-catenin is delivered back to deficient cells, perhaps via SVF-derived sEVs. Many different cell types are known to secrete sEVs containing proteins, lipids, and genetic material; these sEVs serve as a unique mechanism for intercellular communication and have varied and intricate effects on receiving cells [81,82]. This is of interest in the context of adipose biology, as WAT-derived sEVs have widespread effects [83,84], from regulation of hepatic FGF21 expression and glucose handling by the liver [85] to promotion of fatty acid oxidation within melanoma cells, contributing to aggressive tumor cell migration and invasion [86]. sEVs are also secreted from non-adipocyte cell types within WAT, including macrophages [87], endothelial cells [88], and stromal cells [89], and can have profound effects on glucose homeostasis, insulin sensitivity, and inflammation. Recently, Crewe et al. found that endothelial-derived sEVs mediate cross-talk between adipocyte and SVF cell populations [88]. Indeed, they reported a phenomenon strikingly similar to the one we observed in chow-fed *β-cat*^−/−^ mice: despite efficient genetic ablation of *Cav1* (caveolin-1), cav1 protein was readily detectable in deficient adipocytes. Ultimately, this was found to be the result of sEV-mediated trafficking of cav1 protein from surrounding endothelial cells back to *Cav1*^−/−^ adipocytes. In addition to cav1, proteomic analysis of isolated endothelial sEVs identified members of the Wnt signaling pathway, including β-catenin [88]. Thus, a similar mechanism may explain the sustained expression of β-catenin protein in knockout adipocytes. In addition to identification of the cell population contributing elevated β-catenin expression, further studies are required to determine whether β-catenin protein is trafficked back to deficient adipocytes.

To date, studies of Wnt signaling in mature adipocytes have consistently reported that metabolic phenotypes are revealed with long-term HFD. However, conflicting results have emerged: our work in *β-cat*^−/−^ mice fed HFD demonstrated decreased weight gain and fat mass, significantly improved glucose homeostasis, decreased circulating TAG and glucose-stimulated insulin release, and protection from hepatosteatosis. Of note, the metabolic effects observed in our mice were remarkably consistent with data recently reported by Chen et al.; in their study deleting β-catenin from adipocytes, obese *β-cat*^−/−^ mice also exhibited decreased body weight and adiposity, accompanied by improved glucose tolerance, insulin sensitivity, and hepatosteatosis [37]. Additionally, adipocyte-specific *Wntless*^−/−^ mice fed HFD were also characterized by decreased fat mass and protection from glucose intolerance and hepatosteatosis [40]. In contrast, HFD-fed *Tcf7l2*^−/−^ mice demonstrated increased WAT mass, impaired glucose tolerance, and insulin insensitivity [39]. Further, WAT isolated from Tcf7l2 knockout mice had elevated lipogenic gene expression, including *Scd1*. There are many potential reasons for these discordant results, including the generation of alternative Tcf7l2 splice variants [90], compensatory activity of other TCF/LEF transcription factors [18,19], or downstream effects on signaling pathways independent of β-catenin [91]. Nevertheless, it is clear from these studies that Wnt signaling within adipocytes, while complex, plays a critical role in the regulation of lipid metabolism.

One final point of interest is the functional protection from diet-induced obesity and subsequent metabolic dysfunction that loss of adipocyte-specific β-catenin signaling appears to afford mice. Chen et al. found that WAT of HFD-fed *β-cat*^−/−^ mice contained fewer PDGFRα^+^ preadipocytes, indicating that reduced fat mass is caused by decreased hyperplasia [37]. Further, they observed decreased expression of Saa3, a secreted protein that is elevated with obesity and type 2 diabetes and functions to activate macrophages to promote local inflammatory responses [[92], [93], [94]]. Chen et al. proposed the hypothesis that reduced Saa3 in HFD-fed *β-cat*^−/−^ mice leads to less macrophage recruitment and activation and subsequently less PDGFRα^+^ cell proliferation. Indeed, global Saa3-deficient mice are resistant to diet-induced obesity, adipose tissue inflammation, and dyslipidemia [95]. Our studies demonstrated that consistent with macrophage marker expression patterns, *Saa3* was mildly higher in *β-cat*^−/−^ mice maintained on chow diet and was subsequently suppressed in knockout mice with HFD feeding. Thus, it is possible that β-catenin mediates cross-talk between mature adipocytes and surrounding cells through Saa3, but future studies are required to directly answer this question.

In summary, we report that β-catenin-dependent canonical Wnt signaling regulates various metabolic pathways in mature adipocytes, including lipid metabolism. Indeed, β-catenin is required for coordinate regulation of DNL and fatty acid desaturation, partly mediated through the key transcription factors *Srebf1* and *Mlxipl*. Perhaps most interestingly, in chow-fed mice, CD45^-^/CD31^-^ stromal cells respond to adipocyte-specific β-catenin depletion by up-regulating β-catenin and downstream target gene expression to defend canonical Wnt signaling homeostasis within WAT. We contend that this compensatory mechanism may explain the lack of observable phenotypes under standard nutritional conditions in virtually all mouse models that have been developed to interrogate the function of Wnt signaling in adipocytes. Finally, HFD feeding obesity overrides this compensatory mechanism, revealing that *β-cat*^−/−^ mice are protected from diet-induced obesity and metabolic dysfunction. Together, these novel findings underscore the critical importance of Wnt signaling in regulation of glucose and lipid metabolism in mature adipocytes.

## Author contributions

D.P.B. and O.A.M. conceived the project, designed the experiments, and wrote the manuscript. D.P.B performed the majority of the experiments, data analyses, and manuscript preparation. A.N. conducted analyses of the RNA-seq and ChIP-seq data. Z.L. and J. B. D. assisted with the experiments. Z.L., C.A.C., J.H., and B.S.L. assisted with the large-scale animal studies. H.M. and C.N.L. contributed intellectually to the experiments and provided key feedback. O.A.M. supervised the research.

## Appendix A Supplementary data

Supplemental Figure 1 (**A**) Representative immunoblotting of β-catenin protein expression across C57BL/6J mouse tissues (BAT, brown adipose tissue; pWAT, perirenal WAT); adiponectin and laminin included as controls. (**B**) *Dlk1* and *Adipoq* gene expression in the SVF and adipocyte fractions isolated from eWAT and iWAT of C57BL/6J mice (males; n = 5). (**C-D**) *Lep* and *Nkd1* mRNA expression in the SVF and adipocytes isolated from eWAT and iWAT of mice fed an NCD or 10 weeks of HFD (males; n = 5). (**E**) Regulation of *Ctnnb1* gene expression in eWAT and iWAT by nutritional and environmental conditions: fast: 18 h; refed: 6 h after 18 h fast; 30% calorie restriction (CR): 6 weeks; 4 °C cold exposure: 6 h. Each condition normalized to its individual experimental control set to 1 (indicated by dashed line; n = 6). (**F**) *Ctnnb1* gene expression in male and female human tissues; data obtained from the GTEx-RNA-Seq dataset. (**G**) Schematic model for deletion of β-catenin in cultured preadipocytes or adipocytes using adenoviral Cre recombinase. (**H**) *Ctnnb1* gene recombination (n = 2) and β-catenin protein expression (n = 4) in preadipocytes treated with adenoviral GFP or Cre. (**I**) Expression of downstream Wnt target genes in *β-cat*^fl/fl^ and *β-cat*^−/−^ adipocytes treated with vehicle or 3 μM CHIR99021 for 4 h (n = 6). RNA expression normalized to PPIA. Data are presented as mean ± S.D. ∗ indicates significance at p < 0.05. Supplemental Figure 1

Supplemental Figure 2 RNA-seq analyses were performed on *β-cat*^fl/fl^ and *β-cat*^−/−^ preadipocytes under basal conditions or after 4 h of treatment with recombinant Wnt3a (20 ng/ml; n = 4 per group). (**A**) Heat maps of differential gene expression changes in *β-cat*^fl/fl^ and *β-cat*^−/−^ preadipocytes under basal conditions (left panel) and *β-cat*^fl/fl^ cells treated with vehicle or Wnt3a (right panel). (**B**) Gene Set Enrichment Analyses (GSEA) of genes in *β-cat*^fl/fl^ and *β-cat*^−/−^ preadipocytes under basal conditions (top panel) and *β-cat*^fl/fl^ cells treated with vehicle or Wnt3a (bottom panel). (**C-D**) MA plots of gene expression changes following Wnt3a treatment of *β-cat*^fl/fl^ or *β-cat*^−/−^ preadipocytes. (**E**) Venn diagram depicting meta-analysis of gene expression changes in *β-cat*^fl/fl^ or *β-cat*^−/−^ preadipocytes treated with vehicle or Wnt3a for 4 h. (**F**) Expression of *Ctnnb1* and downstream Wnt target genes in *β-cat*^fl/fl^ and *β-cat*^−/−^ preadipocytes treated with vehicle or 20 ng/ml recombinant Wnt3a for 4, 12, or 24 h (n = 6). RNA expression normalized to PPIA. Data are presented as mean ± S.D. ∗ indicates significance at p < 0.05. Supplemental Figure 2

Supplemental Figure 3 (**A**) Lipogenic proteins quantified by densitometry (n = 3). (**B**) Relative proportions of stearic (C18:0) vs vaccenic (C18:1, n-7) and oleic (C18:1, n-9) acid in *β-cat*^fl/fl^ and *β-cat*^−/−^ adipocytes (n = 3). [^14^C]-radiolabel in (**C**) conditioned media vs (**D**) whole cell lysates after indicated incubation times measured by scintillation counting (n = 6). (**E**) Expression of genes related to acetate uptake and activation (n = 6). (**F-G**) Radiolabel incorporation into DAG and PL fractions extracted from *β-cat*^fl/fl^ and *β-cat*^−/−^ adipocytes quantified by scintillation counting (n = 6). (**H**) Linear regression analyses comparing slopes of acetate uptake over time from conditioned media into *β-cat*^fl/fl^ and *β-cat*^−/−^ adipocytes. (**I**) Linear regression analyses comparing slopes of radiolabel incorporation over time into TAG fractions of *β-cat*^fl/fl^ and *β-cat*^−/−^ adipocytes. (**J**) Gene expression in *β-cat*^fl/fl^ and *β-cat*^−/−^ adipocytes treated with adenovirus expressing GFP, ChREBP, or SREBP1c for 72 h (1 x 10^5^ viral particles/ml; n = 4). RNA expression normalized to PPIA. Data are presented as mean ± S.D. ∗ indicates significance at p < 0.05. Supplemental Figure 3

Supplemental Figure 4 (**A**) Representative traces and quantification of daily running distance in chow-fed male 23-week-old *β-cat*^fl/fl^ and *β-cat*^−/−^ mice given access to voluntary exercise wheels for six weeks. (**B**) Growth curve of 28-week-old female *β-cat*^fl/fl^ and *β-cat*^−/−^ mice. (**C**) Body composition of 16-week-old *β-cat*^fl/fl^ and *β-cat*^−/−^ mice on an NCD. (**D**) Glucose and insulin tolerance tests in 16- and 19-week-old *β-cat*^fl/fl^ and *β-cat*^−/−^ mice, respectively. (**E**) Blood glucose concentrations in randomly fed and 16 h fasted mice. Serum concentrations of (**F**) insulin, (**G**) adiponectin, and (**H**) TAG in 28-week-old mice. (**I**) Tissue weights at time of sacrifice. (**J**) Representative histological images of H&E-stained tissues from female *β-cat*^fl/fl^ and *β-cat*^−/−^ mice fed an NCD for 28 weeks; 200x magnification; scale bar, 100 μm. Data in B-J from female mice, n = 6 per group. Data are presented as mean ± S.D. ∗ indicates significance at p < 0.05. Supplemental Figure 4

Supplemental Figure 5 (**A**) Representative histological images of H&E-stained proximal tibia (regulated bone marrow adipose tissue) of male and female *β-cat*^fl/fl^ and *β-cat*^−/−^ mice fed an NCD for 28 weeks (male: n = 8; female: n = 6). (**B**) μCT analyses of tibial trabecular bone volume fraction (Tb. BV/TV) and trabecular bone mineral density (Tb. BMD). (**C**) Representative histological images of H&E-stained distal tibia (constitutive bone marrow adipose tissue) of male and female *β-cat*^fl/fl^ and *β-cat*^−/−^ mice. (**D**) μCT analyses of mid-tibial cortical bone area (Ct. BA/TA) and cortical bone mineral density (Ct. BMD). Histological images are shown at 100x magnification; scale bar, 50 μm. Data are presented as mean ± S.D. ∗ indicates significance at p < 0.05. Supplemental Figure 5

Supplemental Figure 6 (**A-D**) Lipogenic gene expression in whole eWAT, iWAT, liver, or isolated eWAT adipocytes of *β-cat*^fl/fl^ and *β-cat*^−/−^ mice (n = 8). (**E**) Expression of immune, endothelial, and stromal cell markers in the SVF isolated from eWAT of *β-cat*^fl/fl^ and *β-cat*^−/−^ mice (n = 6). (**F**) Expression of inflammatory markers in whole eWAT of *β-cat*^fl/fl^ and *β-cat*^−/−^ mice (n = 6; n = 10). (**G-H**) Expression of cell markers and Wnt target genes in SVF sub-populations isolated by FACS analysis (3 mice per sample; n = 3 samples). RNA expression normalized to PPIA. Data are presented as mean ± S.D. ∗ indicates significance at p < 0.05. Supplemental Figure 6

Supplemental Figure 7 (**A**) Daily food intake of male *β-cat*^fl/fl^ and *β-cat*^−/−^ mice fed an HFD for 24 weeks. (**B**) ITT area under the curve analysis of male *β-cat*^fl/fl^ and *β-cat*^−/−^ HFD-fed mice. (**C**) Pancreas weights at time of sacrifice of HFD-fed mice (n = 5, n = 11). (**D-E**) mRNA expression of adipokines in eWAT and iWAT of HFD-fed mice (n = 8). (**F**) Immunoblotting and densitometry quantification of UCP1 and β-catenin protein expression in BAT isolated from HFD-fed *β-cat*^fl/fl^ and *β-cat*^−/−^ mice (n = 4). Data are presented as mean ± S.D. ∗ indicates significance at p < 0.05. Supplemental Figure 7
