# Supplementary figures and images for: Wnt/β-catenin signaling regulates adipose tissue lipogenesis and adipocyte-specific loss is rigorously defended by neighboring stromal-vascular cells
Source: Mol Metab. 2020 Sep 9;42:101078. doi: 10.1016/j.molmet.2020.101078 (PMC7554252; doi:10.1016/j.molmet.2020.101078)

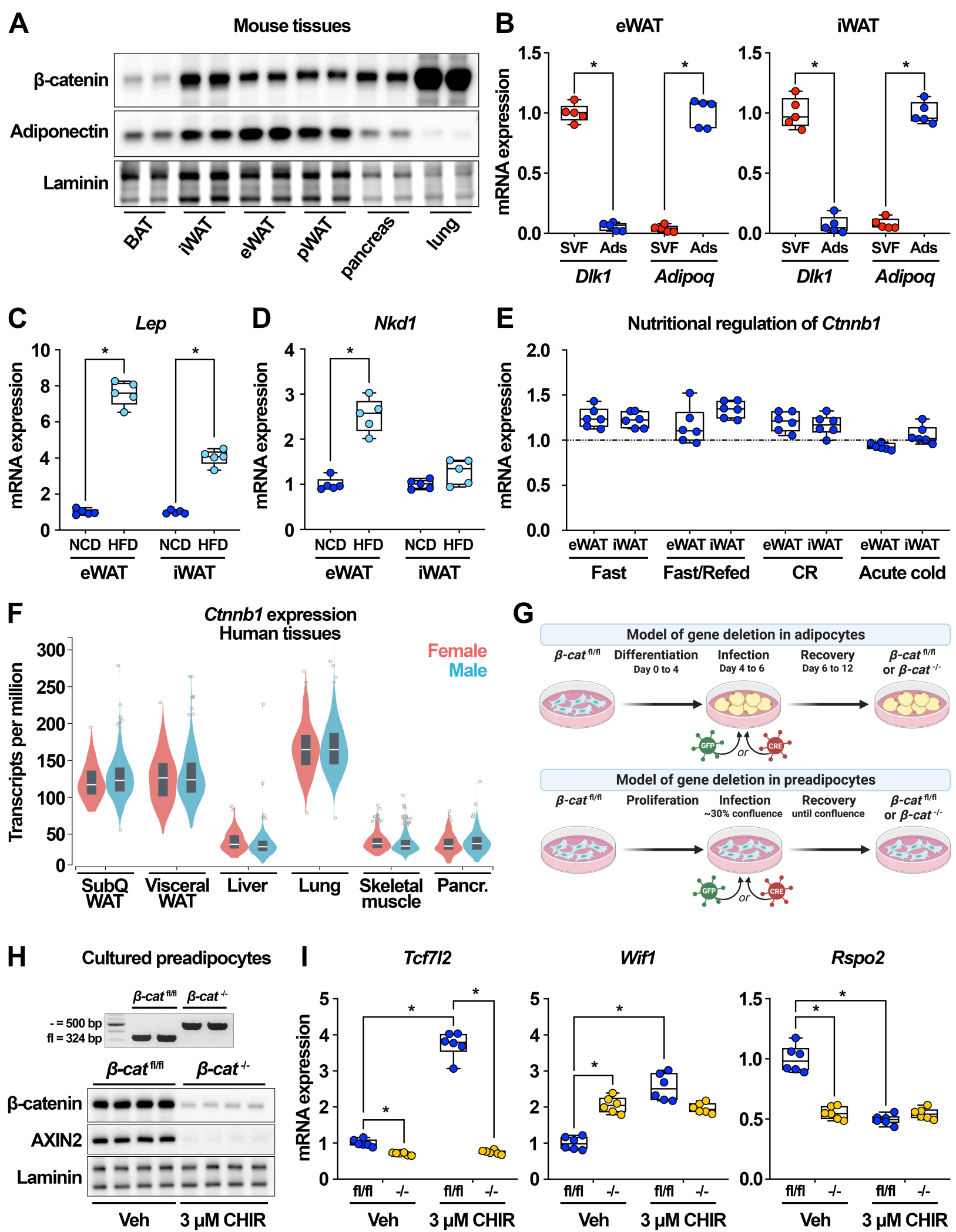

Supplement: Supplemental Figure 1 — (A) Representative immunoblotting of β-catenin protein expression across C57BL/6J mouse tissues (BAT, brown adipose tissue; pWAT, perirenal WAT); adiponectin and laminin included as controls. (B) Dlk1 and Adipoq gene expression in the SVF and adipocyte fractions isolated from eWAT and iWAT of C57BL/6J mice (males; n = 5). (C-D) Lep and Nkd1 mRNA expression in the SVF and adipocytes isolated from eWAT and iWAT of mice fed an NCD or 10 weeks of HFD (males; n = 5). (E) Regulation of Ctnnb1 gene expression in eWAT and iWAT by nutritional and environmental conditions: fast: 18 h; refed: 6 h after 18 h fast; 30% calorie restriction (CR): 6 weeks; 4 °C cold exposure: 6 h. Each condition normalized to its individual experimental control set to 1 (indicated by dashed line; n = 6). (F) Ctnnb1 gene expression in male and female human tissues; data obtained from the GTEx-RNA-Seq dataset. (G) Schematic model for deletion of β-catenin in cultured preadipocytes or adipocytes using adenoviral Cre recombinase. (H) Ctnnb1 gene recombination (n = 2) and β-catenin protein expression (n = 4) in preadipocytes treated with adenoviral GFP or Cre. (I) Expression of downstream Wnt target genes in β-catfl/fl and β-cat−/− adipocytes treated with vehicle or 3 μM CHIR99021 for 4 h (n = 6). RNA expression normalized to PPIA. Data are presented as mean ± S.D. ∗ indicates significance at p < 0.05. [file mmc1.pdf]

# Preadipocytes

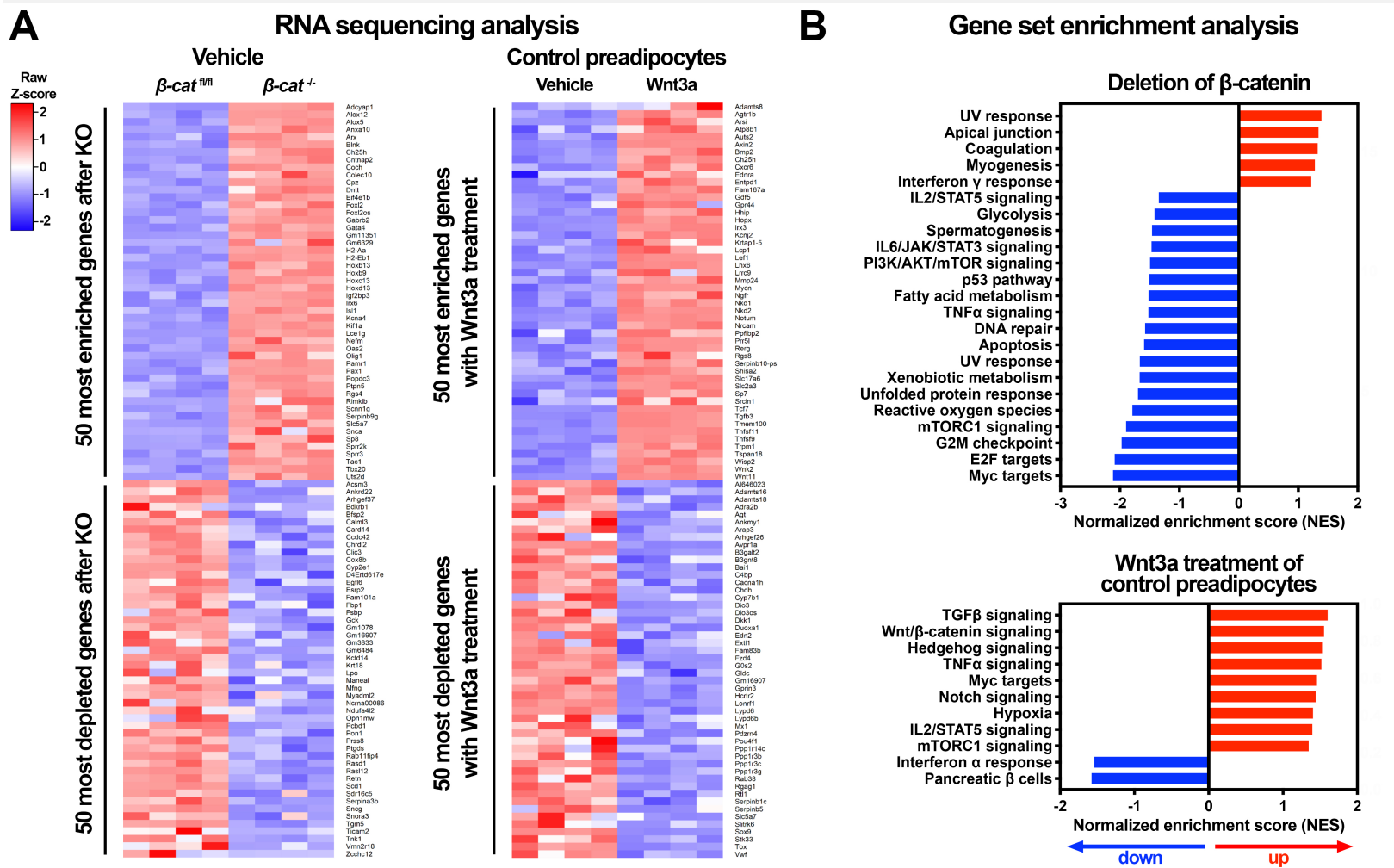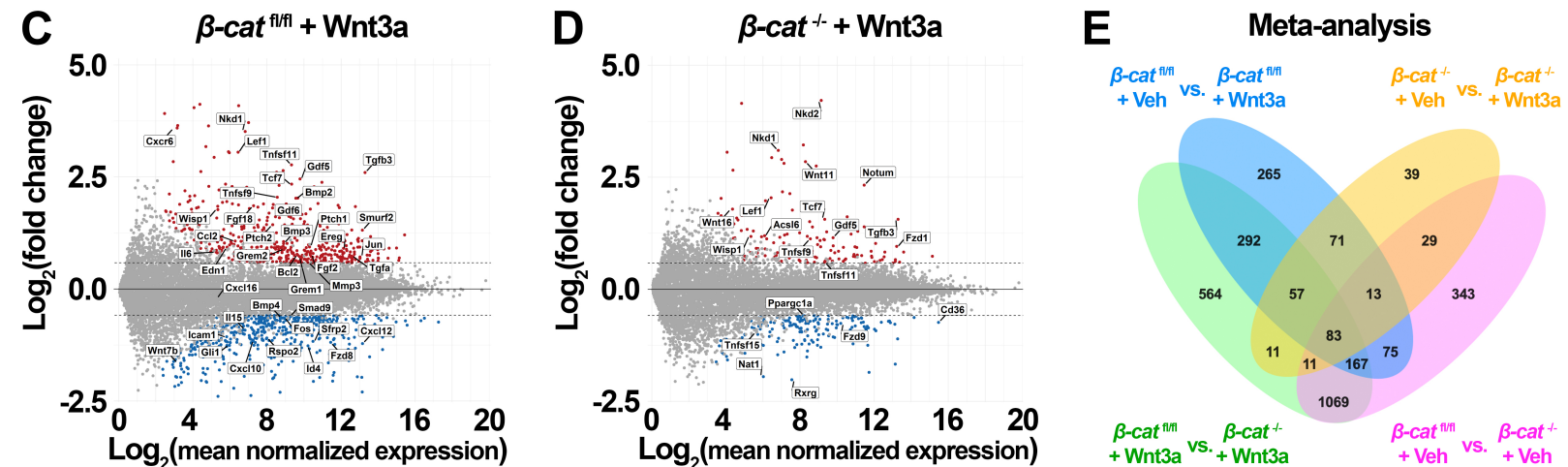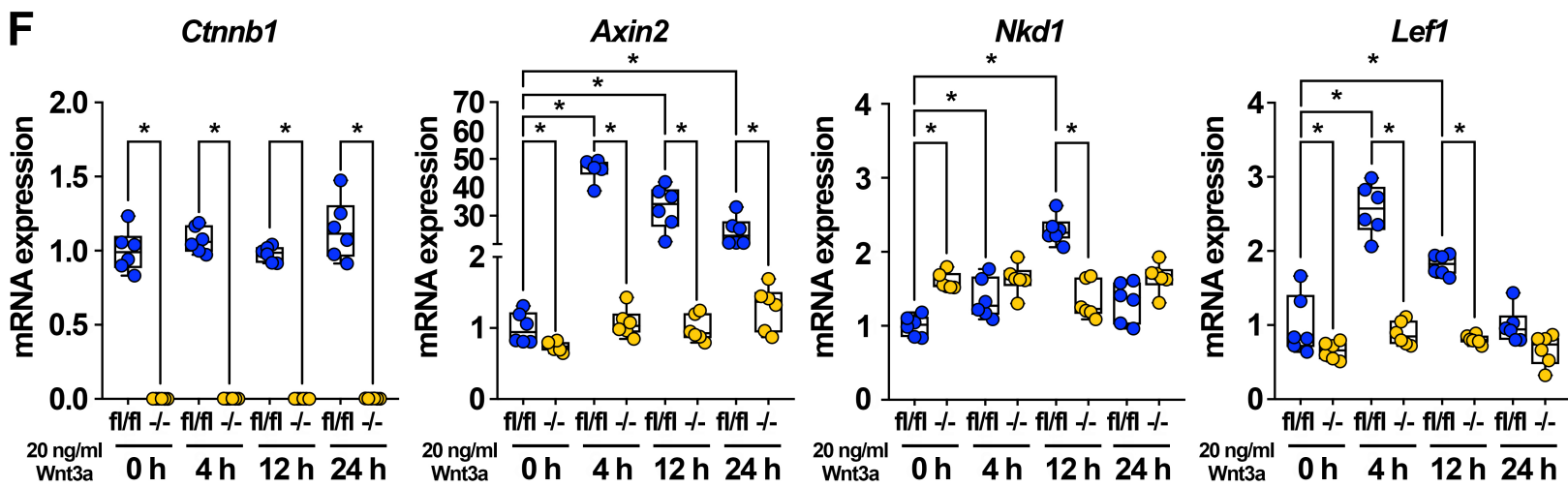

Supplement: Supplemental Figure 2 — RNA-seq analyses were performed on β-catfl/fl and β-cat−/− preadipocytes under basal conditions or after 4 h of treatment with recombinant Wnt3a (20 ng/ml; n = 4 per group). (A) Heat maps of differential gene expression changes in β-catfl/fl and β-cat−/− preadipocytes under basal conditions (left panel) and β-catfl/fl cells treated with vehicle or Wnt3a (right panel). (B) Gene Set Enrichment Analyses (GSEA) of genes in β-catfl/fl and β-cat−/− preadipocytes under basal conditions (top panel) and β-catfl/fl cells treated with vehicle or Wnt3a (bottom panel). (C-D) MA plots of gene expression changes following Wnt3a treatment of β-catfl/fl or β-cat−/− preadipocytes. (E) Venn diagram depicting meta-analysis of gene expression changes in β-catfl/fl or β-cat−/− preadipocytes treated with vehicle or Wnt3a for 4 h. (F) Expression of Ctnnb1 and downstream Wnt target genes in β-catfl/fl and β-cat−/− preadipocytes treated with vehicle or 20 ng/ml recombinant Wnt3a for 4, 12, or 24 h (n = 6). RNA expression normalized to PPIA. Data are presented as mean ± S.D. ∗ indicates significance at p < 0.05. [file mmc2.pdf]

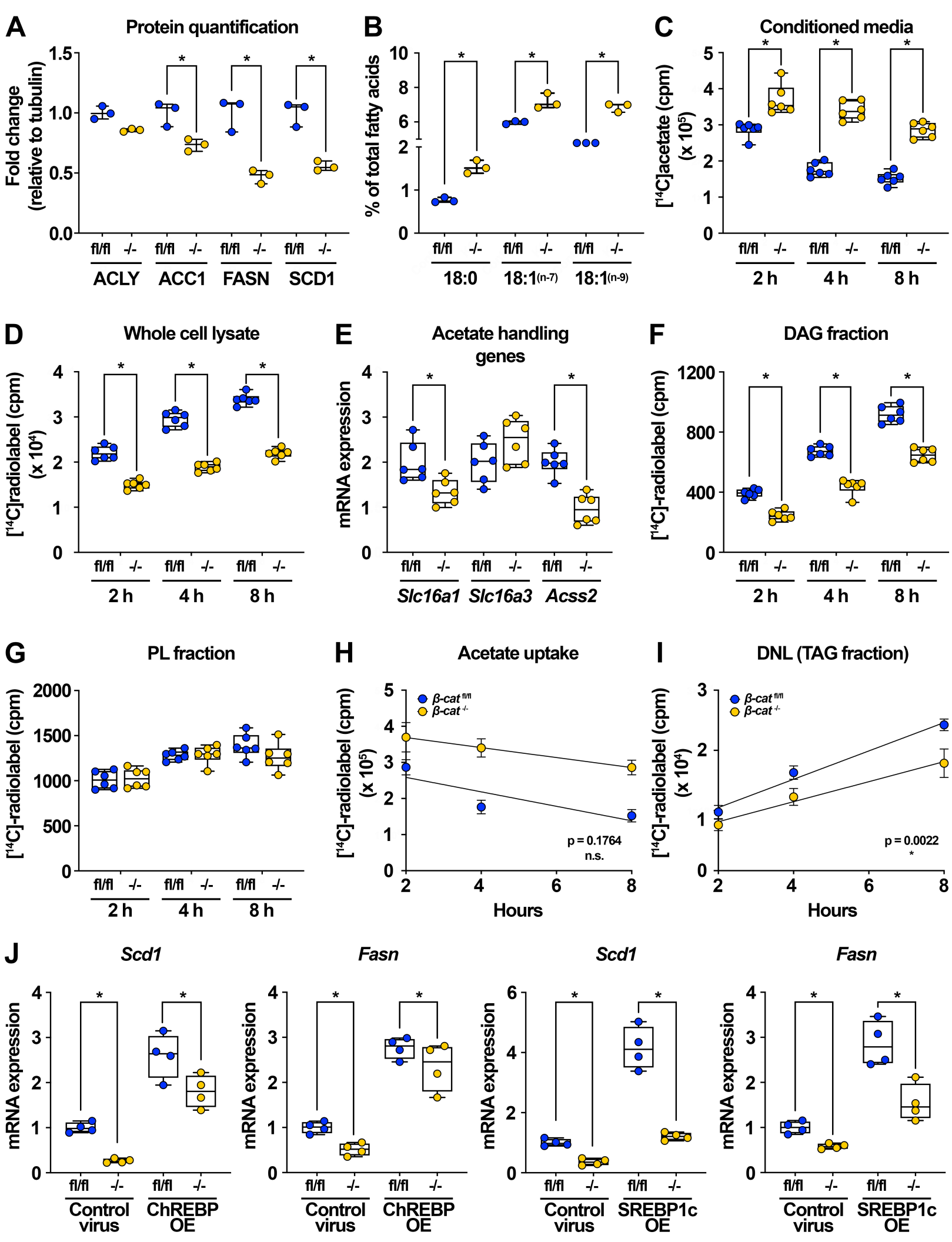

Supplement: Supplemental Figure 3 — (A) Lipogenic proteins quantified by densitometry (n = 3). (B) Relative proportions of stearic (C18:0) vs vaccenic (C18:1, n-7) and oleic (C18:1, n-9) acid in β-catfl/fl and β-cat−/− adipocytes (n = 3). [14C]-radiolabel in (C) conditioned media vs (D) whole cell lysates after indicated incubation times measured by scintillation counting (n = 6). (E) Expression of genes related to acetate uptake and activation (n = 6). (F-G) Radiolabel incorporation into DAG and PL fractions extracted from β-catfl/fl and β-cat−/− adipocytes quantified by scintillation counting (n = 6). (H) Linear regression analyses comparing slopes of acetate uptake over time from conditioned media into β-catfl/fl and β-cat−/− adipocytes. (I) Linear regression analyses comparing slopes of radiolabel incorporation over time into TAG fractions of β-catfl/fl and β-cat−/− adipocytes. (J) Gene expression in β-catfl/fl and β-cat−/− adipocytes treated with adenovirus expressing GFP, ChREBP, or SREBP1c for 72 h (1 x 10^5^ viral particles/ml; n = 4). RNA expression normalized to PPIA. Data are presented as mean ± S.D. ∗ indicates significance at p < 0.05. [file mmc3.pdf]

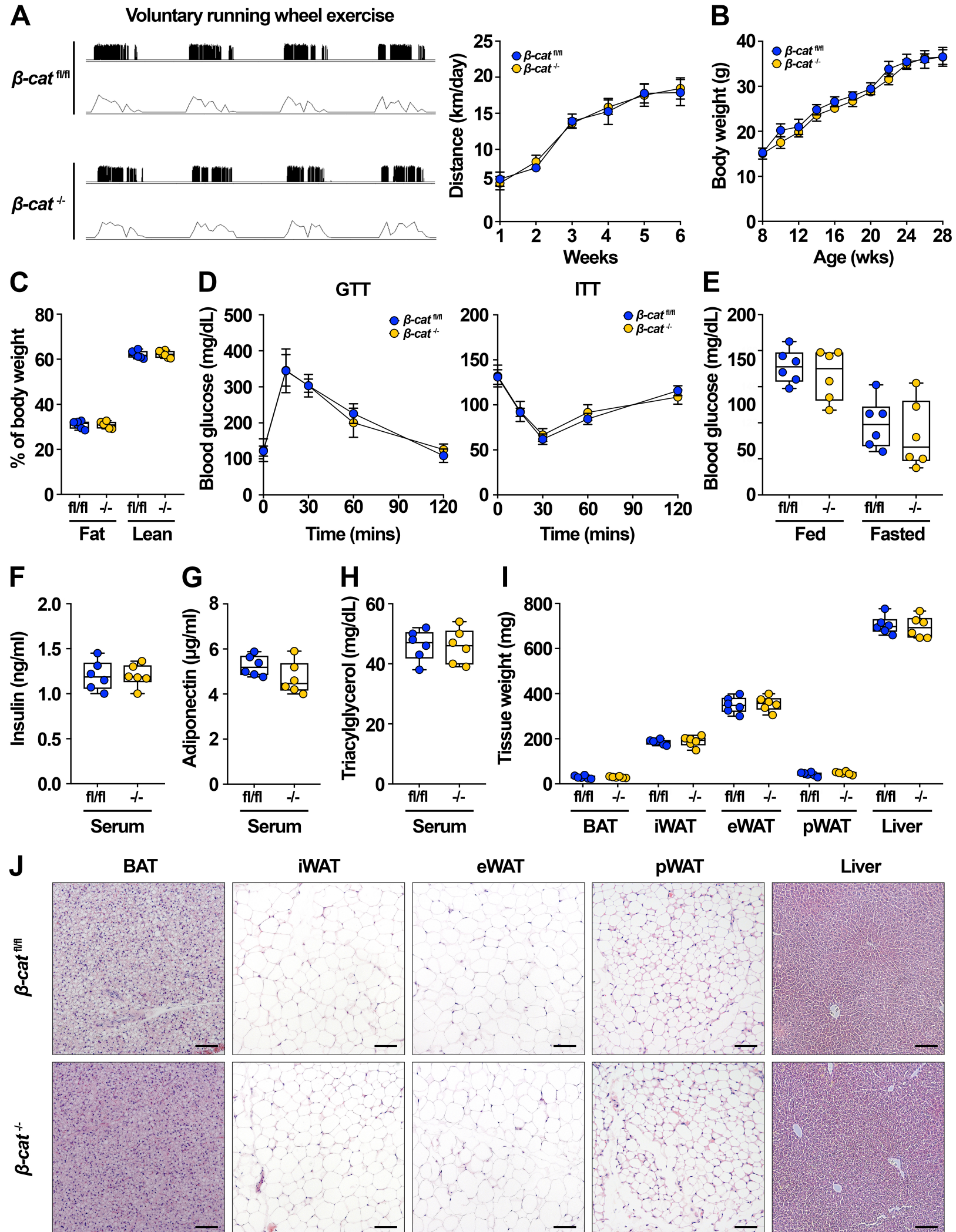

Supplement: Supplemental Figure 4 — (A) Representative traces and quantification of daily running distance in chow-fed male 23-week-old β-catfl/fl and β-cat−/− mice given access to voluntary exercise wheels for six weeks. (B) Growth curve of 28-week-old female β-catfl/fl and β-cat−/− mice. (C) Body composition of 16-week-old β-catfl/fl and β-cat−/− mice on an NCD. (D) Glucose and insulin tolerance tests in 16- and 19-week-old β-catfl/fl and β-cat−/− mice, respectively. (E) Blood glucose concentrations in randomly fed and 16 h fasted mice. Serum concentrations of (F) insulin, (G) adiponectin, and (H) TAG in 28-week-old mice. (I) Tissue weights at time of sacrifice. (J) Representative histological images of H&E-stained tissues from female β-catfl/fl and β-cat−/− mice fed an NCD for 28 weeks; 200x magnification; scale bar, 100 μm. Data in B-J from female mice, n = 6 per group. Data are presented as mean ± S.D. ∗ indicates significance at p < 0.05. [file mmc4.pdf]

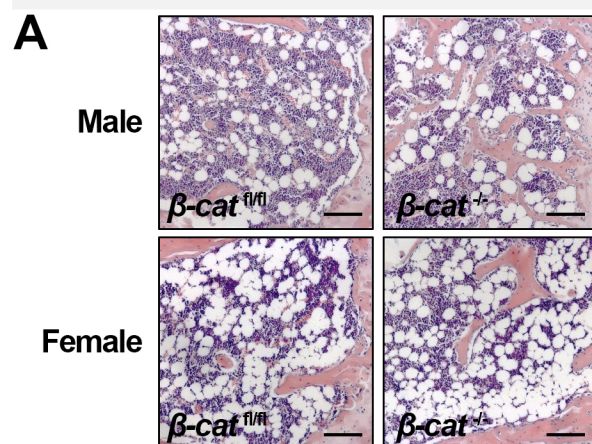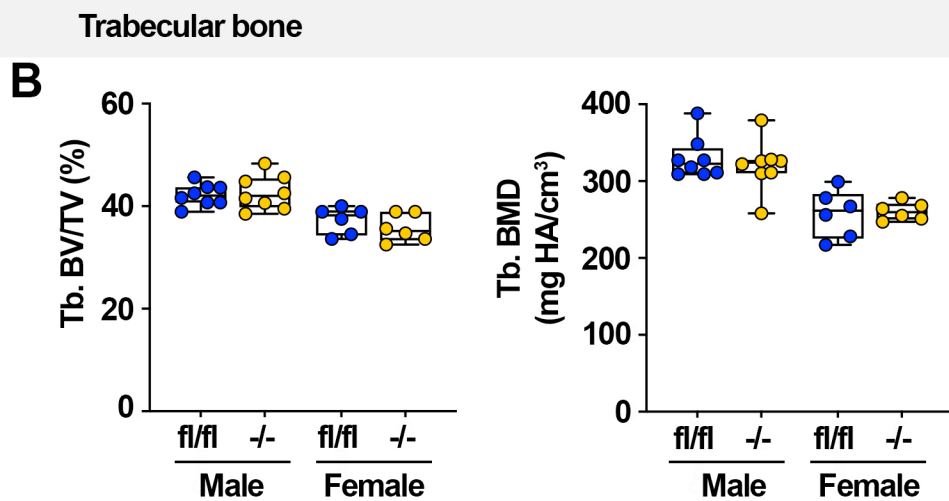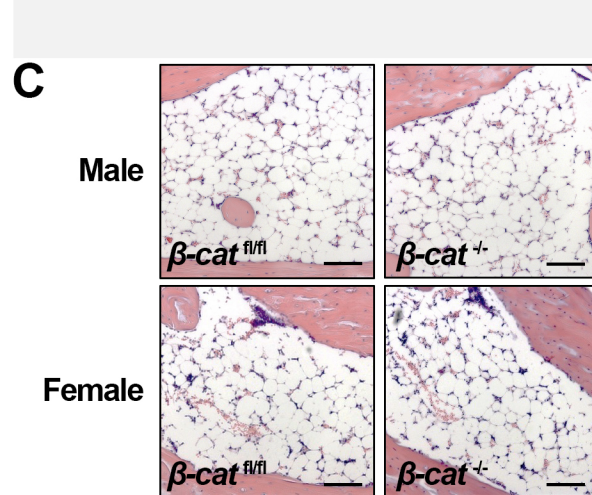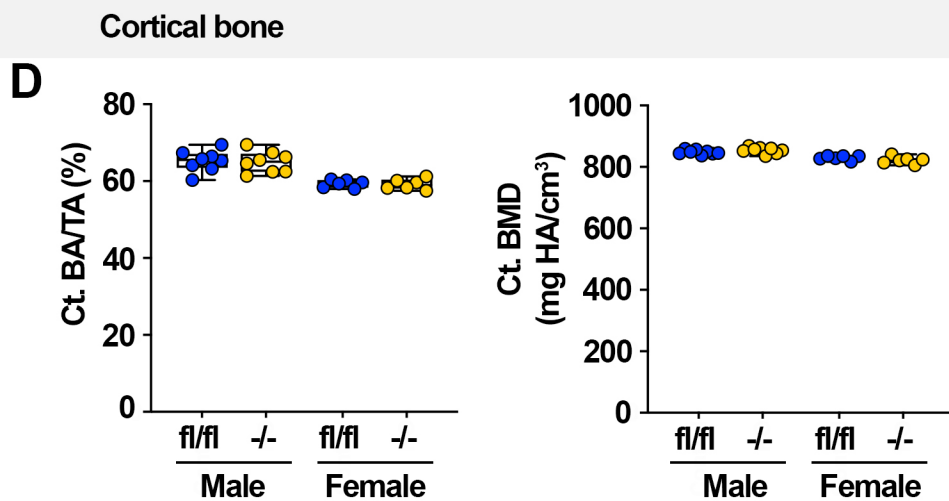

Supplement: Supplemental Figure 5 — (A) Representative histological images of H&E-stained proximal tibia (regulated bone marrow adipose tissue) of male and female β-catfl/fl and β-cat−/− mice fed an NCD for 28 weeks (male: n = 8; female: n = 6). (B) μCT analyses of tibial trabecular bone volume fraction (Tb. BV/TV) and trabecular bone mineral density (Tb. BMD). (C) Representative histological images of H&E-stained distal tibia (constitutive bone marrow adipose tissue) of male and female β-catfl/fl and β-cat−/− mice. (D) μCT analyses of mid-tibial cortical bone area (Ct. BA/TA) and cortical bone mineral density (Ct. BMD). Histological images are shown at 100x magnification; scale bar, 50 μm. Data are presented as mean ± S.D. ∗ indicates significance at p < 0.05. [file mmc5.pdf]

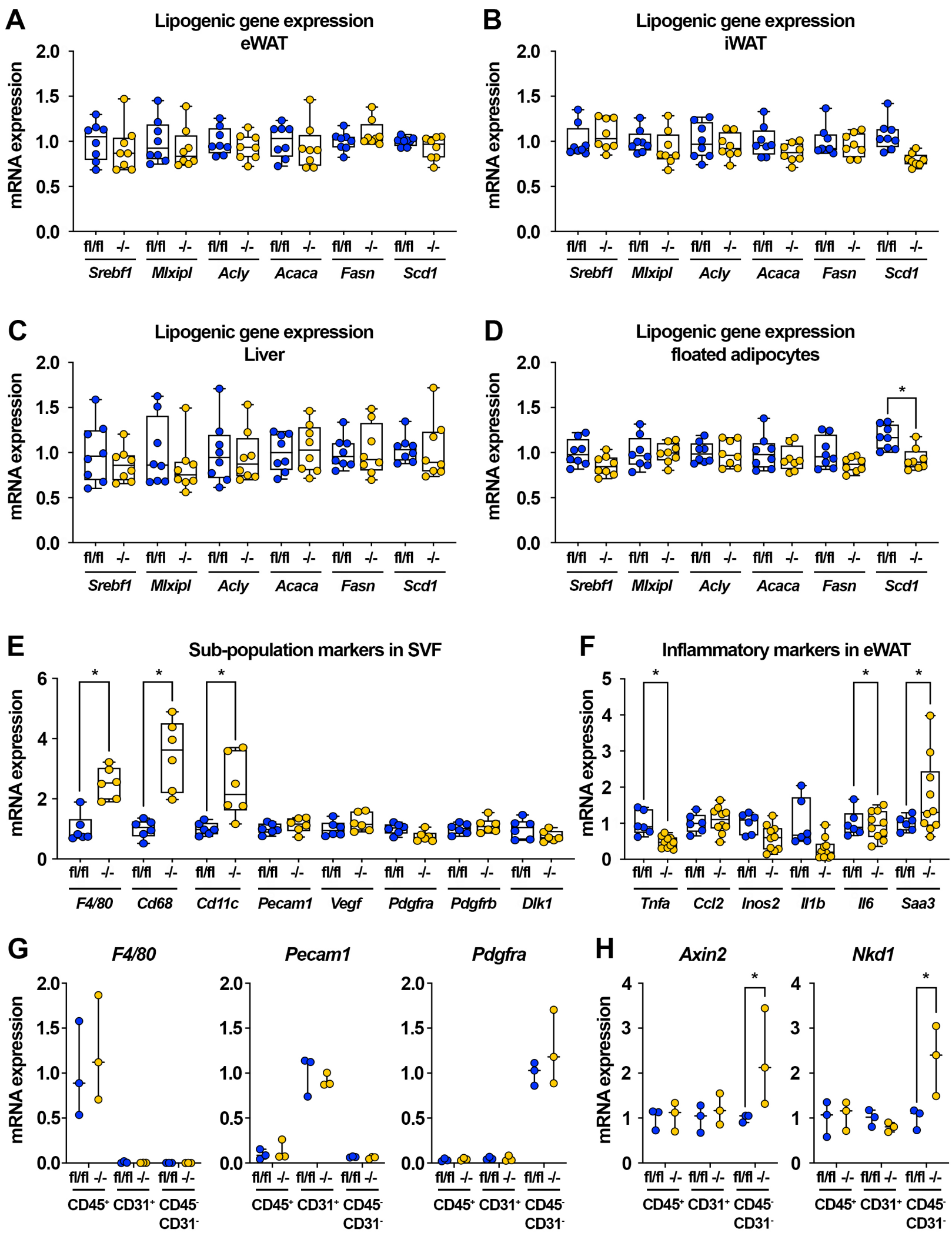

Supplement: Supplemental Figure 6 — (A-D) Lipogenic gene expression in whole eWAT, iWAT, liver, or isolated eWAT adipocytes of β-catfl/fl and β-cat−/− mice (n = 8). (E) Expression of immune, endothelial, and stromal cell markers in the SVF isolated from eWAT of β-catfl/fl and β-cat−/− mice (n = 6). (F) Expression of inflammatory markers in whole eWAT of β-catfl/fl and β-cat−/− mice (n = 6; n = 10). (G-H) Expression of cell markers and Wnt target genes in SVF sub-populations isolated by FACS analysis (3 mice per sample; n = 3 samples). RNA expression normalized to PPIA. Data are presented as mean ± S.D. ∗ indicates significance at p < 0.05. [file mmc6.pdf]

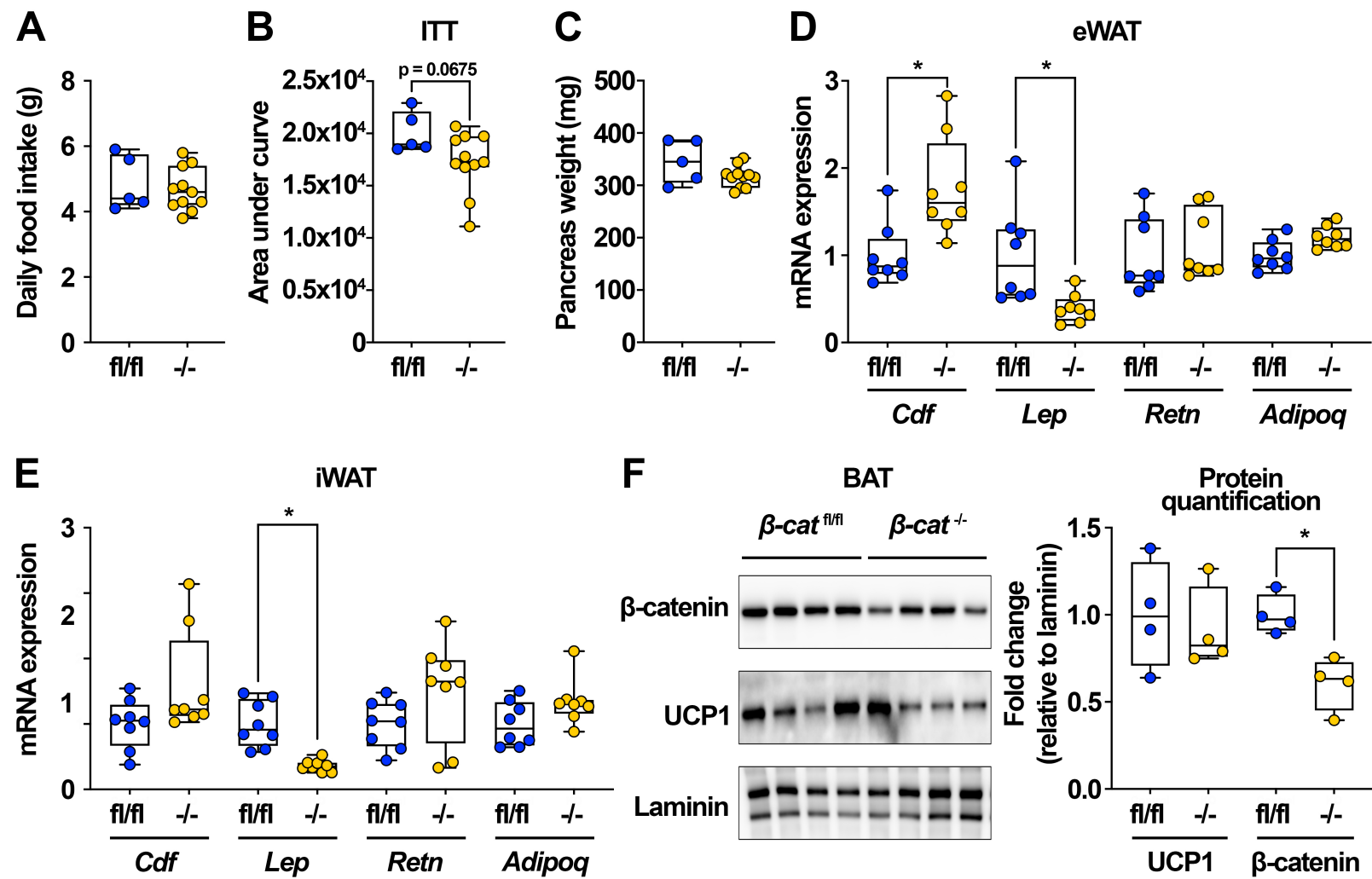

Supplement: Supplemental Figure 7 — (A) Daily food intake of male β-catfl/fl and β-cat−/− mice fed an HFD for 24 weeks. (B) ITT area under the curve analysis of male β-catfl/fl and β-cat−/− HFD-fed mice. (C) Pancreas weights at time of sacrifice of HFD-fed mice (n = 5, n = 11). (D-E) mRNA expression of adipokines in eWAT and iWAT of HFD-fed mice (n = 8). (F) Immunoblotting and densitometry quantification of UCP1 and β-catenin protein expression in BAT isolated from HFD-fed β-catfl/fl and β-cat−/− mice (n = 4). Data are presented as mean ± S.D. ∗ indicates significance at p < 0.05. [file mmc7.pdf]
