# Supplementary material for: Wnt/β-catenin signaling regulates adipose tissue lipogenesis and adipocyte-specific loss is rigorously defended by neighboring stromal-vascular cells
Source: Mol Metab. 2020 Sep 9;42:101078. doi: 10.1016/j.molmet.2020.101078 (PMC7554252; doi:10.1016/j.molmet.2020.101078)
Supplement: Multimedia component 8 [file mmc8.docx]

**Supplemental Table 1: qPCR primer sequences**

| **Gene** | **Forward sequence** | **Reverse sequence** |
| --- | --- | --- |
| *Acaca* | TAAACCAGCACTCCCGATTC | CCATCCTGTAAGCCAGAGAT |
| *Acly* | AGGTGGCCCCAACTATCAAGAG | CGCTGGCATTAAGGAGGAAGTT |
| *Acss2* | CCTTCAGCCCCACACTCACAG | CAATGGGGCCAATCTTTTCTCT |
| *Adipoq* | GCCAAACACCGATTGGGGT | GGCTCCAAATCTCCTTGGTAGTT |
| *Axin2* | CCCAGAGCCCGTCACAGC | GATCGCCAAGGGGAAGGTAAC |
| *Ccl2* | GCTTCTGGGCCTGCTGTTC | TGCTGCTGGTGATCCTCTTGTA |
| *Cd11c* | TGCTGCTGCTGGCTATCATCA | TGGGCGGGTTCAAAGACG |
| *Cd68* | CTTCCCACAGGCAGCACAG | AATGATGAGAGGCAGCAAGAGG |
| *Cdf* | CTGCGACGTGGCTGGTTG | AGTGTCCCTGCGGTTGCTCT |
| *Cepba* | TCACTGGTCAACTCCAGCAC | TGGACAAGAACAGCAACGAG |
| *Ctnnb1* | TGCTCATCCCACTAATGTCCAG | AGCTTCCTTTTTGGAAAGCTGAT |
| *Dlk1* | AGTGCGAAACCTGGGTGTC | GCCTCCTTGTTGAAAGTGGTCA |
| *Elovl6* | GCACCCGAACTAGGTGACAC | CCCCAGCGACCATGTCTTT |
| *Elovl7* | CTGGGGTACAGGTTACTCGTT | CGGCATGATCGTATGATGGAAGA |
| *F4/80* | CTTTGGCTATGGGCTTCCAGTC | GCAAGGAGGACAGAGTTTATC |
| *Fasn* | GTCGTCTGCCTCCAGAGC | GTTGGCCCAGAACTCCTGTA |
| *Gpat3* | CGGATTATCCCTGGGTATCTCG | CGAAGTCCCTTCCTCGAAGAC |
| *Id2* | GCATCCTGTCCTTGCAGGCATCTG | AGTCCAGGCCGGAGAACGACA |
| *Il1b* | AAGAGCTTCAGGCAGGCAGTAT | CCACGGGAAAGACACAGGTAG |
| *Il6* | CCAGAGATACAAAGAAATGAT | ACTCCAGAAGACCAGAGGAAAT |
| *Inos2* | AATCTTGGAGCGAGTTGTGG | CAGGAAGTAGGTGAGGGCTTG |
| *Lef1* | GCCACCGATGAGATGATCCC | TTGATGTCGGCTAAGTCGCC |
| *Lep* | GCGGGATCAGGTTTTGTGGT | TTCCCGGTCTCTTCAGTCTTCC |
| *Lxra* | TCAGCATCTTCTCTGCAGACCGG | TCATTAGCATCCGTGGGAACA |
| *Lxrb* | AAGCTGGTGAGCCTGCGC | CGGCAGCTTCTTGTCCTG |
| *Mlxipl* | CCAGCCTCAAGGTGAGCAAA | CATGTCCCGCATCTGGTCA |
| *Nkd1* | AAGGCTGCCCGTCCATCA | TACCCCCATTCCACCCTAAGTC |
| *Pdgfra* | GACACGCTCCGGGTATCATCTT | CCTCGGCCTCGGGAACTT |
| *Pdgfrb* | gtgacagactacctctttgg | ctacatctcccagtgtctcc |
| *Pecam1* | AAGCCAACAGCCATTACGGT | AGCCTTCCGTTCTCTTGGTG |
| *Pparg* | CCAGAGCATGGTGCCTTCGC | TTCCGAAGTTGGTGGGCCAGA |
| *Ppia* | CACCGTGTTCTTCGACATCA | CAGTGCTCAGAGCTCGAAACT |
| *Retn* | CCCCTTCCCCTTCCTCTTTG | GCCCTGGGTGCCTCTGGT |
| *Rspo2* | CTGCGCTCGGCTGCTTCTA | CACTGCCTTCTGACCCTGGAG |
| *Saa3* | GCCTGGGCTGCTAAAGTCATC | AGGCCAGCAGGTCGGAAGT |
| *Scd1* | CGTGGGTTGGCTGCTTGTG | CAGGAGGCCGGGCTTGTAGT |
| *Slc16a1* | TCCTCATCATCGCGGGTATCTA | TGCTGGCCTCGTCCTCTTTT |
| *Slc16a3* | AACGCCTGCTAGACCTGAGTGT | GTGGGCCTGGCAAAGATGT |
| *Srebf1* | GGAGCCATGGATTGCACATT | GCTTCCAGAGAGGAGGCCAG |
| *Tcf7l2* | CCCACCGCCCGAACCTAT | ACGGCGAACGAGCATCCTT |
| *Tnfa* | CATCTTCTCAAAATTCGAGTGACAA | TGGGAGTAGACAAGGTACAACCC |
| *Vegf* | CTGTGCAGGCTGCTGTAACG | GTTCCCGAAACCCTGAGGAG |
| *Wif1* | GCGGGGCAGGCAGAATAC | CGAGACACGGGAAACCAACTT |
| *Wnt10b* | GCTGCGGATGGAAGGGTAGT | AGGGCTGGGCTGGGAGAT |
| *Wnt16* | CCGGCTCCTGTGCTGTGAA | AATGGGGGTCTGCCTCTGGT |

**Supplemental Table 2: Immunoblotting antibodies**

| **Protein** | **Vendor** | **Catalog #** |
| --- | --- | --- |
| β-catenin | Abcam | ab16051 |
| ACC1 | Cell Signaling Technology | 3662 |
| ACLY | Proteintech | 15421-1-AP |
| Adiponectin | Sigma-Aldrich | A6354 |
| AXIN2 | Proteintech | 20540-1-AP |
| C/EBPa | Cell Signaling Technology | 2295 |
| ChREBP | Novus Biologicals | NB400-135 |
| ELOVL7 | Abcam | ab106633 |
| FASN | Abcam | ab22759 |
| Laminin | Novus Biologicals | NB300-144 |
| Perilipin | Abcam | ab3526 |
| PPARg | Millipore | MAB3872 |
| Pref1 | R&D Systems | AF8277 |
| SCD1 | Cell Signaling Technology | 2438 |
| SREBP1c | Santa Cruz Biotechnology | sc-13551 |
| Tubulin | Invitrogen | MA1-80017 |
| UCP1 | Alpha Diagnostic International | UCP11-A |
| WISP2 | LifeSpan Biosciences | LS-C349158-50 |

**Supplemental methods**

***Voluntary running wheel exercise***

Mice were single-housed for one week prior to beginning exercise studies. They were then given free access to running wheels (Columbus Instruments, Columbus, OH, USA) for voluntary exercise. Daily running totals were calculated from wheel revolutions collected at 1 min intervals for a total of six weeks.

***Micro-computed tomography (μCT) analysis of bone***

Tibia were harvested and fixed in 10% neutral buffered formalin for 24 h, rinsed with water, and stored at 4°C in Sorensen’s phosphate buffer at pH 7.4 prior to μCT analysis. For the analysis, each bone was placed in a 19-mm diameter tube and its length was scanned using a μCT system as previously described [40] (μCT100 Scanco Medical, Bassersdorf, Switzerland). Analyses of bone parameters were performed with the manufacturer’s evaluation software using a threshold of 280 for cortical bone and 180 for trabecular bone.

***Human gene expression data***

Human *Ctnnb1* gene expression data were obtained from the publicly available Genotype-Tissue Expression (GTEx) database.
